# Methods for the purification and detection of single nucleotide *KRAS* mutations on extrachromosomal circular DNA in human plasma

**DOI:** 10.1002/cam4.6385

**Published:** 2023-08-21

**Authors:** Lasse Bøllehuus Hansen, Sandra Fugl Jakobsen, Egija Zole, Julie Boertmann Noer, Li Tai Fang, Sefa Alizadeh, Julia Sidenius Johansen, Marghoob Mohiyuddin, Birgitte Regenberg

**Affiliations:** ^1^ Department of Biology University of Copenhagen Copenhagen Denmark; ^2^ Roche Sequencing Solutions Belmont California USA; ^3^ Department of Oncology Copenhagen University Hospital Herlev Denmark; ^4^ Department of Medicine Copenhagen University Hospital Herlev Denmark; ^5^ Department of Clinical Medicine, Faculty of Health and Medical Sciences University of Copenhagen Copenhagen Denmark

**Keywords:** circular DNA, eccDNA, *KRAS* mutations, liquid biopsy, phenol/chloroform DNA extraction, plasmids

## Abstract

**Backgrounds:**

Despite recent advances, many cancers are still detected too late for curative treatment. There is, therefore, a need for the development of new diagnostic methods and biomarkers. One approach may arise from the detection of extrachromosomal circular DNA (eccDNA), which is part of cell‐free DNA in human plasma.

**Aims:**

First, we assessed and compared two methods for the purification of eccDNA from plasma. Second, we tested for an easy diagnostic application of eccDNA liquid biopsy‐based assays.

**Materials & Methods:**

For the comparison we tested a solid‐phase silica purification method and a phenol/chloroform method with salt precipitation.

For the diagnostic application of eccDNA we developed and tested a qPCR primer‐based SNP detection system, for the detection of two well‐established cancer‐causing *KRAS* mutations (G12V and G12R) on circular DNA. This investigation was supported by purifying, sequencing, and analysing clinical plasma samples for eccDNAs containing *KRAS* mutant alleles in 0.5 mL plasma from 16 pancreatic ductal adenocarcinoma patients and 19 healthy controls.

**Results:**

In our method comparison we observed, that following exonuclease treatment a lower eccDNA yield was found for the phenol/chloroform method (15.7%–26.7%) compared with the solid‐phase purification approach (47.8%–65.9%).

For the diagnostic application of eccDNA tests, the sensitivity of the tested qPCR assay only reached ~10^−3^ in a background of 10^5^ wild type (wt) *KRAS* circular entities, which was not improved by general amplification or primer‐based inhibition of wt *KRAS* amplification. Furthermore, we did not detect eccDNA containing *KRAS* in any of the clinical samples.

**Discussion:**

A potential explanation for our inability to detect any *KRAS* mutations in the clinical samples may be related to the general low abundance of eccDNA in plasma.

**Conclusion:**

Taken together our results provide a benchmark for eccDNA purification methods while raising the question of what is required for the optimal fast and sensitive detection of SNP mutations on eccDNA with greater sensitivity than primer‐based qPCR detection.

## Graphical abstract

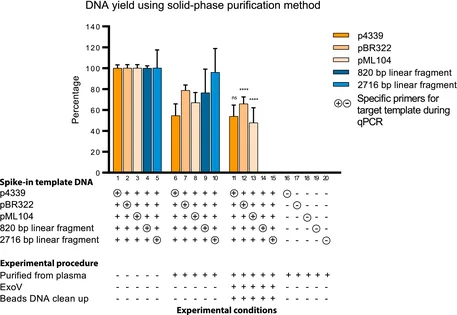

## 1 INTRODUCTION

Despite recent advances in the detection and diagnosis of cancer, many cancers are still diagnosed late, resulting in low patient survival rates. This is particularly true for pancreatic ductal adenocarcinoma (PDAC) and lung cancers (LCs), for which more than half of all patients are diagnosed at a late stage.^1^ Unfortunately, many late‐stage tumors are considered unresectable, and only palliative and life‐extending treatments can be offered.^2^ The primary reason for the late diagnosis is an asymptomatic early‐stage disease progression and the lack of general screening programs, as such, there is a large need for novel diagnostic markers and methods capable of detecting early‐stage cancers.^3^ The establishment of an early detection regime can also help guide clinicians in their diagnosis and enable them to initiate improved treatment at a stage where the patient can be cured. Due to their easy accessibility and high amount of biomolecules and circulating cells, liquid biopsies are strong screening candidates for general testing regimes.^4^ In plasma, the analysis of cell‐free DNA (cfDNA), circulating cancer cells, and exosomes have already proven their potential as biomarkers for PDAC detection.^5^, ^6^ cfDNA, in particular, has been thoroughly investigated, as plasma DNA can be purified in a few steps and subsequently analyzed for specific markers using PCR or DNA sequencing. However, disease‐relevant cfDNA is often fragmented and is only present in low amounts in plasma,^7^ especially at the early stages of PDAC progression,^8^, ^9^, ^10^ which limits their application as easily applicable biomarkers.

A potential alternative to linear cfDNA is cell‐free extrachromosomal circular DNA (eccDNA). eccDNA offers the same options for analysis as linear ctDNA but is expected to have a lower degradation rate than linear DNA (lDNA).^11^ Kumar et al. have shown that eccDNA exists in the plasma, and that eccDNA from four LC patients can be used to direct treatment efforts.^12^ These results suggest a potential for the usage of eccDNA from plasma as a biomarker in cancer.^12^, ^13^, ^14^, ^15^, ^16^ As such, there is a relevant need for efficient laboratory practices and methodologies that provide high‐quality eccDNA and establish eccDNA as a biomarker.

Currently the most well‐established biomarker for pancreatic cancer is the sialylated Lewis blood‐group antigen also known as carbohydrate antigen 19–9 (CA 19–9). It is used for diagnosis, prognosis, and to monitor response to treatment. However, it lacks sensitivity due to part of the population not producing the Lewis blood‐group antigen. It also lacks sensitivity, since it is also detected in patients with other gastrointestinal diseases.^17^

One alternative set of biomarkers that has been suggested for use in pancreatic cancer diagnostics is the *KRAS* mutations.^18^, ^19^, ^20^ *KRAS* mutations are some of the most frequent genetic modifications in PDAC, with an occurrence frequency of more than 90%.^21^, ^22^ The *KRAS* gene (Kirsten rat sarcoma virus) belongs to the *RAS* gene family and is involved in cell proliferation, differentiation, and apoptosis.^23^ *KRAS* mutations have also been studied as potential biomarkers in cfDNA by using deep next‐generation sequencing.^24^, ^25^ However, these methods are not yet well established in the clinic.

The current standard for the purification of cfDNA in human biological fluids is the QIAamp Circulating Nucleic Acid Kit (Qiagen), a commercially available silica column‐based solid‐phase purification method,^26^, ^27^ which relies on vacuum column DNA binding and the usage of carrier RNA. In this paper, we have provided an alternative phenol/chloroform‐based purification method and compared its ability to purify intact eccDNA with the solid‐phase purification method. We assessed the plasma purification efficiency by measuring spiked‐in plasmids, which were quantified using a DNA intercalating fluorophore assay and polymerase chain reaction (PCR)‐based techniques. Our results have enabled us to establish an efficient general method for the purification of intact eccDNA with high yield and purity that can be applied to detect mutant alleles on eccDNA in plasma.

## 2 MATERIALS AND METHODS

### 2.1 Plasmids

All plasmids (Figures S1 and S4) were maintained in *Escherichia coli* and purified using a plasmid DNA purification kit (NucleoBond Xtra Midi, Macherey‐Nagel, DE, #740410.50), in accordance with the manufacturer's protocol. Spike in size, mix ratios, and concentrations are detailed in Tables 1 and 2.

**TABLE 1:** Spike‐in content, 10 μL^a^.

Plasmid or linear gene fragment	Size, bp	Concentration, ng	Copies
p4339	5315	0.1165	2 × 10^7^
pBR322	4361	0.2464	2 × 10^7^
pML104	11,240	0.0956	2 × 10^7^
Linear fragment of *scGNP1*	337	5	‐
Linear fragment of *scAGP1*	820	5	‐
Linear fragment of *scACT1*	1409	5	‐
Linear fragment containing *scBCP1*	2716	5	‐

^a^ The content of 10 μL of spike‐in used to determine DNA purification yields (Figure S1, maps and sequences).

**TABLE 2:** Quantity of spike‐in mixes^a^.

	Wild‐type *KRAS* plasmid, copies	Mutant *KRAS* plasmid, copies	Mutant allele mix frequency, %
Mix 1	0	100,000	100
Mix 2	100,000	100,000	50
Mix 3	100,000	10,000	10
Mix 4	100,000	1000	1.0
Mix 5	100,000	100	0.1
Mix 6	100,000	10	0.01
Mix 7	100,000	0	0

^a^ The content of 1‐μL spike‐in mix used for *KRAS* single nucleotide polymorphism (SNP) detection (Figure S4, maps and sequences).

### 2.2 Patient samples

Pretreatment plasma samples (~0.5 mL) from 16 Stage III‐IV PDAC patients were obtained from the prospective Danish BIOPAC study “BIOmarkers in patients with PAncreatic Cancer” (BIOPAC [NCT03311776]).^28^ The BIOPAC study has been approved by the Danish Ethics Committee (VEK, j.nr. KA‐20060113 and H‐16043715) and the Danish Data Protection Agency (j.nr. 2006‐41‐6848, 2012‐58‐0004; HGH‐2015‐027; I‐Suite j.nr. 03960; and PACTICUS P‐2020‐834). The study was conducted in accordance with the Declaration of Helsinki. Signed informed consent was obtained from each participant. Patients were on average 69 years old, 50% were male and 81% had Stage IV disease. Plasma samples from (19) healthy donors were obtained from Aalborg University Hospital. Healthy donors were on average 63 years old and 32% were male.

### 2.3 Phenol/chloroform purification and salt precipitation of DNA from plasma

Pooled plasma from healthy human donors (Innovative Research, SKU: IPLAWBK2E500ML) was thawed from −80°C at room temperature. For each sample preparation, 1 mL of plasma was placed in a 2 mL tube and mixed with 10 μL of spike‐in DNA in accordance with Table 1. Sixty‐four micro liters of 20% sodium dodecyl sulfate (SDS) (G Biosciences, #DG092) was added to each sample as well as 22 μL of Proteinase K (20 mg/mL). Samples were incubated at 37°C for 1 h, then denatured at 95–98°C for 5 min and incubated on ice for 5 min. The samples were then divided into two tubes; phenol/chloroform/isoamyl alcohol (25:24:1, pH‐7.9) (Invitrogen, #15593031) was added in a 1:1 sample volume ratio and mixed by carefully inverting the tubes at least six times. The samples were then centrifuged at 14,000 rpm for 10 min at room temperature in an Eppendorf 5453 MiniSpin plus. The aqueous top phase containing the DNA was transferred to a clean 2‐mL tube with a pointed bottom. GlycoBlue Coprecipitant (Thermo Fisher Scientific, #AM9515) (1:300), 3 M sodium acetate (1:10, pH‐5.2) (Sigma‐Aldrich), and 100% ethanol (2.5× the total volume, including GlycoBlue and sodium acetate) were added to the aqueous phase in the respective volumes specified to precipitate DNA. The samples were mixed and incubated at −20°C for 1 h before being centrifuged at 17,000 rcf for 30 min at 4°C. The supernatants were discarded and the pellets were washed in 500 μ ice‐cold 70% ethanol. Each sample was then centrifuged at 17,000 rcf for 10 min at 4°C. The supernatants were discarded, and the pellets were dried until the ethanol had evaporated. Each pellet was then resuspended in 50 μL 10 mM Tris–HCl (pH‐8). Samples were left at room temperature to dissolve for 30 min to 1 h. The two halves of the divided samples were then combined to a total volume of 100 μL.

For hospital samples, the above protocol was used with the modification that proteinase K digestion was excluded and the final volume was 50 μL.

### 2.4 Column‐based, solid‐phase (QIAamp) purification of DNA from plasma

Pooled plasma from healthy human donors (Innovative Research, SKU: IPLAWBK2E500ML) was thawed from −80°C at room temperature. For each sample preparation; 1 mL of plasma was placed in a 1.5 mL tube and mixed with 10 μL of spike‐in DNA in accordance with Table 1. DNA was purified with the QIAamp Circulating Nucleic Acid Kit (Qiagen, DE, #55114) according to the manufacturer's protocol for purification from 1, 2, or 3 mL plasma.

### 2.5 Removal of linear DNA with exonuclease V

Exonuclease V kit (RecBCD, New England BioLabs, #M0345S) was used to remove lDNA. Each DNA sample was mixed with 1 mM ATP, 1X NEBuffer 4, and 24U of exonuclease V. The samples were then incubated at 37°C in a heat block for 1 h, following which the exonuclease V was heat‐inactivated at 70°C for 30 min.

### 2.6 DNA clean‐up using AMPure XP beads

Agencourt AMPure XP beads (Beckman Coulter, #A63882) were equilibrated to room temperature and vortexed thoroughly before being added to each DNA sample in a 1.8× beads‐to‐volume ratio (starting volumes of 80 and 100 μL). Samples were incubated at room temperature for 5 min and then placed on a DynaMag‐2 magnet (Thermo Fisher Scientific, #12321D) for at least 2 min. The solution was aspirated, and the beads were washed with 400 μL 70% ethanol twice. All ethanol was aspirated, and each tube was removed from the magnet. Tris–HCl (10 mM, pH‐8.0) was added to the beads for DNA elution. The samples were then incubated at 50°C for 5 min and placed at the magnet for at least 2 min. The eluted DNA was then transferred to a clean tube, and the elution process was repeated. The final clean‐up product consisted of the combined two eluates for each sample.

### 2.7 Quantification of DNA yield by qPCR and qubit

The yields of the plasmids and linear fragments after DNA purification from plasma and *KRAS* mutations were quantified by qPCR (Tables 3 and 4).

**TABLE 3:** Oligos for qPCR of the plasmids and linear fragments were as follows.

Plasmids or linear fragments	Primer sequence	Plasmid or fragment length (bp)/qPCR fragment length (bp)
p4339	5′‐TGCCCTGCCCCTAATCAGTA‐3′ 5′‐CTGGGCAGATGATGTCGAGG‐3′	5315 bp/149 bp
pBR322	5′‐CCTCTTGCGGGATATCGTCC‐3′ 5′‐AGAACGGGTGCGCATAGAAA‐3′	4361 bp/99 bp
pML104	5′‐TCCGGTTCCCAACGATCAAG‐3′ 5′‐AGTGATAACACTGCGGCCAA‐3′	11,240 bp/116 bp
AGP1	5′‐GTTTTGGGTTTGCAGTCGCT‐3′ 5′‐ATCCGGGTTCACAGATGTCG‐3′	820 bp/127 bp
BCP1	5′‐CGGTGGTAACCCAGAAGTTGA‐3′ 5′‐TGTGGTGGTTGGGGAACCTA‐3′	2716 bp/130 bp

**TABLE 4:** *KRAS* plasmids wt and mutations at position 12.

Mutation	Primer sequence	Plasmid length (bp)/qPCR fragment length (bp
*KRAS* G12V sequence in pUC57	5′‐CACGTCTGCAGTCAACTGGA‐3′ 5′‐ACTCTTGCCTACGCCAA‐3′	3651 bp/236 bp
*KRAS* G12R sequence in pUC57	5′‐CACGTCTGCAGTCAACTGGA‐3′ 5′‐ACTCTTGCCTACGCCTCT‐3′	3651 bp/236 bp
*KRAS* G12D sequence in pUC57	5′‐CACGTCTGCAGTCAACTGGA‐3′ 5′‐ACTCTTGCCTACGCCAT‐3′	3651 bp/236 bp
*KRAS* wt dd‐primer reverse primer	5′‐ACTCTTGCCTACGCCACddC‐3′	3651 bp/236 bp

All reactions were run in triplicate in a QuantStudio 7 Flex qPCR machine (Applied Biosystems) in 10 μL reactions with 1 μL template DNA, 150 nM of each primer (300 nM of the *KRAS* wild type (wt) 3′‐dideoxy nucleotide reverse primer (dd‐primer)), and 5 μL SYBR Green PCR master mix (Thermo Fisher Scientific, #4309155). Prior to each run, the reaction plate was spun down in a Heraeus Megafuge 8 centrifuge (Thermo Fisher Scientific). The PCR reaction conditions were 10 min at 95°C, followed by 40 cycles of 15 s at 95°C and 30 s at 60°C. Melting curves were used to verify reaction specificity. DNA concentration was also measured with Qubit (Thermo Fisher Scientific, #Q32851) in accordance with the manufacturer's protocol.

### 2.8 Optimization of qPCR

A potential optimization of the qPCR detection of all three *KRAS* mutants (G12D, G12V, and G12R) through increased annealing temperatures was assessed (Program: 10 min at 95°C, followed by 40 cycles of 15 s at 95°C and 30 s at either 62 or 63°C).

The relative effect of increased dd‐primer primer for the detection of the G12D mutant was tested by reducing the forward and reverse primer concentration by 33% (from 150 nM to 100 nM) while increasing the dd‐primer wt primer by 33% (from 300 to 400 nM).

### 2.9 Rolling circle amplification

EccDNA was amplified using TruePrime Rolling Circle Amplification Kit (4basebio, #390100). A circular DNA template of 15 μL was prepared in accordance with the manufacturer's protocol and incubated at 30°C for 48 h. The polymerase was then heat‐inactivated at 65°C for 10 min.

### 2.10 Sequencing

Amplified eccDNA from patient samples was fragmented to a mean size of 400 bp on a Bioruptor Pico instrument (Diagenode). Libraries were constructed using a standard protocol for multiplexed dual‐indexed Illumina libraries.^29^ Libraries were sequenced on a NovaSeq 6000 instrument (Illumina) at 2 × 150 bp paired‐end sequencing. Sequencing depth was on average 68.7 million reads per library.

### 2.11 eccDNA mapping

Reads from sequencing in fastq format were aligned to the GRCh38 reference genome using BWA mem (v0.7.17‐r1188) with standard options. eccDNA were called and mapped using a bioinformatics pipeline as previously published.^30^ If their presence was supported by only structural read variants, they were classified as low confidence. If the eccDNA interval had 95% coverage or more, they were classified as high confidence. eccDNA intervals' overlap with genes were annotated with gene coordinates from RefSeq. Variants in eccDNA were called with GATK (version 4.1.1.0) HaplotypeCaller. The variants were annotated with information from ClinVar (v09/2022)^31^ using the biomaRt R package (v2.52.0).^32^, ^33^

### 2.12 Statistics

The product yield of our two purification methods, phenol/chloroform purification with salt precipitation and QIAamp Circulating Nucleic Acid Kit, was compared using a two‐tailed *t*‐test in excel (Microsoft Excel 2016). An unpaired *t*‐test was performed using GraphPad Prism version 8.0.0 for Windows to compare the detection of *KRAS* SNP's with and without the use of Φ29 amplification. An unpaired multiple *t*‐test, corrected using the Bonferroni approach, was performed using Graphpad Prism 8.0.0 to compare the detection of *KRAS* SNP's with and without the use of a *KRAS* wt dd‐primer primer. The level of significance was set at *p* < 0.05 prior to the Bonferroni correction.

## 3 RESULTS

### 3.1 Experimental setup

We tested the efficiency of two purification methods for eccDNA extraction from plasma and compared their yields and sensitivity for the detection of *KRAS* mutations on synthesized circular DNA. The first method is based on the QIAamp Kit, a standardized commercial solid‐phase DNA purification method. The second is an in‐house‐prepared method for the purification of DNA from plasma, based on phenol/chloroform purification and salt precipitation. We tested the DNA yield at two stages of the purification (1) directly after primary DNA purification and (2) after 1 h of enzymatic removal of lDNA from total DNA by exonuclease treatment to evaluate the two DNA purification methods in regards to general and eccDNA yields. Plasma purification yields were for each method determined through qPCR of spiked in plasmids and lDNA. We designed plasmids containing a part of the *KRAS* gene allele, including two of its most common mutation sites and wt for codon G12, to evaluate whether SNPs on circular DNA in plasma can be detected. To get an optimal signal from these mutant alleles, we designed primer pairs in which the 3′ end of the reverse primer covered the common mutation site.

### 3.2 Circular DNA purification from plasma with a solid‐phase method

**FIGURE 1:**
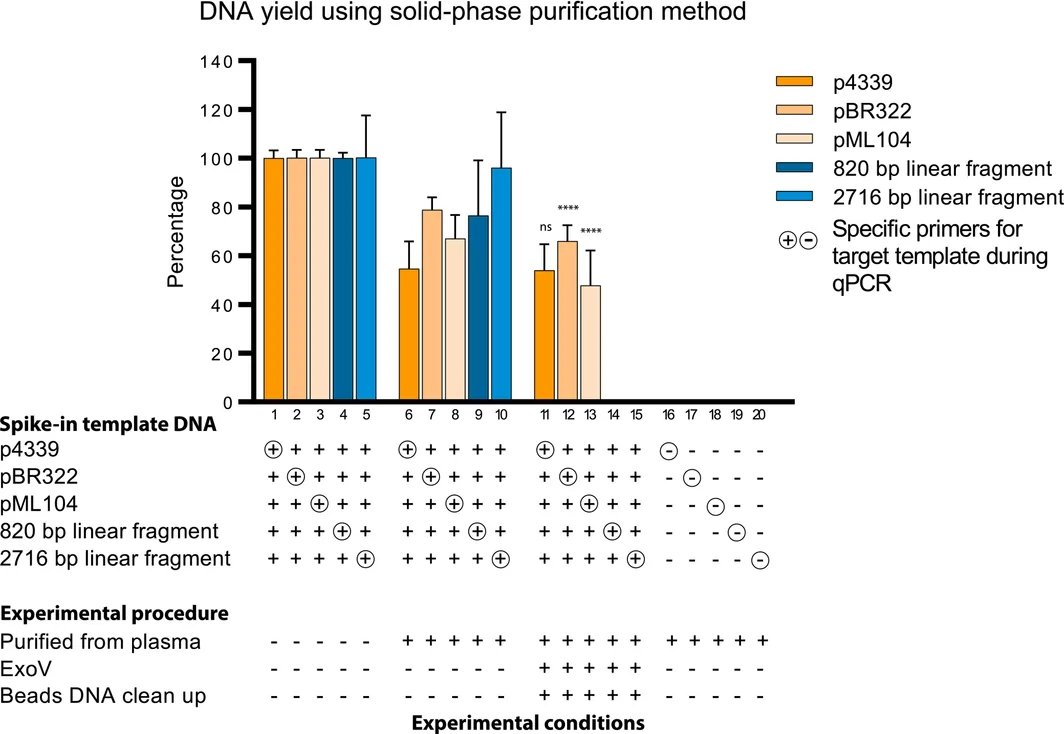
DNA purification yields from plasma using the solid‐phase purification method (QIAamp, Qiagen). DNA was detected by qPCR, and the first five bars represent measured spike‐in baseline controls, which have been set to 100%. Three independent purifications from plasma were conducted and measured in triplicates by qPCR, and the purified DNA yields were calculated from the ct values relative to each sample control (Bars 1–5). Bars 1–5 (positive baseline controls, nine data points/bar), Bars 6–15 (samples purified from plasma, 27 data points/bar), and Bars 16–20 (negative controls, nine data points/bar). Circular DNA is presented using orange bars, and linear DNA fragments are presented using blue bars. All data are presented as mean ± standard deviation.

The solid‐phase silica coloumn‐based purification method (QIAamp Kit) provided average yields in the range of 67% (±13.2%) of the spiked‐in plasmids and 86.3% (±24.3%) of the spiked‐in lDNA, indicating a significantly greater purification yield for lDNA compared with the plasmids (*p* ≤ 0.0001) (Figure 1). The yield for p4339 did not show a significant drop following lDNA digestion (from 54.6% [±11.0%] to 54.0% [±10.6%], *p* > 0.05); however, we observed a significant drop for both pBR322 (from 78.8% [±5.0%] to 65.9% [±6.4%], *p* ≤ 0.0001) and pML104 (from 67.0% [±9.5%] to 47.8% [±14.1%], *p* ≤ 0.0001) (Figure 1). The low variation in the pre‐ and post‐endonuclease digestion numbers suggests that the methodology is capable of purifying eccDNA without causing extensive linearizing damage to the circular DNA population. All spiked‐in lDNA was close to undetectable following 1 h of exonuclease digestion, supporting an efficient lDNA removal.

### 3.3 Circular DNA purification from plasma with phenol/chloroform purification and salt precipitation

**FIGURE 2:**
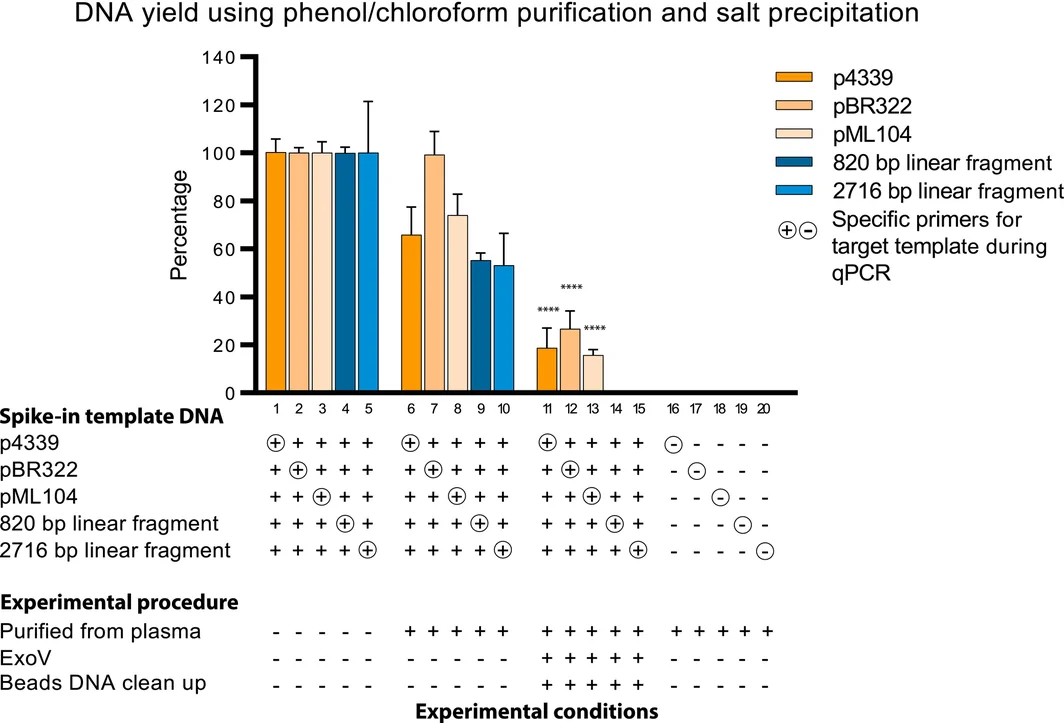
DNA purification yield from plasma using phenol/chloroform purification and salt precipitation. DNA was detected by qPCR, and the first five bars represent measured spike‐in baseline controls, which have been set to 100%. Three independent purifications from plasma were conducted and measured in triplicates by qPCR, and the purified DNA yields were calculated from the ct values relative to each sample control (Bars 1–5). Bars 1–5 (positive baseline controls, nine data points/bar), Bars 6–15 (samples purified from plasma, 27 data points/bar), and Bars 16–20 (negative controls, nine data points/bar). Circular DNA is presented using orange bars, and linear DNA fragments are presented using blue bars. All data are presented as mean ± standard deviation.

Next, we tested our in‐house prepared phenol/chloroform purification with the salt precipitation DNA purification method. This method provided yields ranging from 79.5% (±17.2%) of the spiked‐in plasmid to 54.2% (±9.6%) of the spiked‐in lDNA, indicating a significantly greater purification yield for eccDNA compared with the lDNA (*p* ≤ 0.0001) (Figure 2). The plasmid yield following exonuclease digestion was significantly reduced for all three circular DNA elements, p4339 (from 65.9% [±11.4%] to 18.7% [±8.2%], *p* ≤ 0.0001), pBR322 (from 99.2% [±9.4%] to 26.7% [±7.4%], *p* ≤ 0.0001), and pML104 (from 74.1% [±8.5%] to 15.7% [±2.3%], *p* ≤ 0.0001) (Figure 2). This suggests that the circular DNA was susceptible to the exonuclease digestion after phenol/chloroform purification. All spiked‐in lDNA was close to undetectable following 1 h of exonuclease digestion, supporting an efficient lDNA removal.

### 3.4 Comparing the two purification methods

**FIGURE 3:**
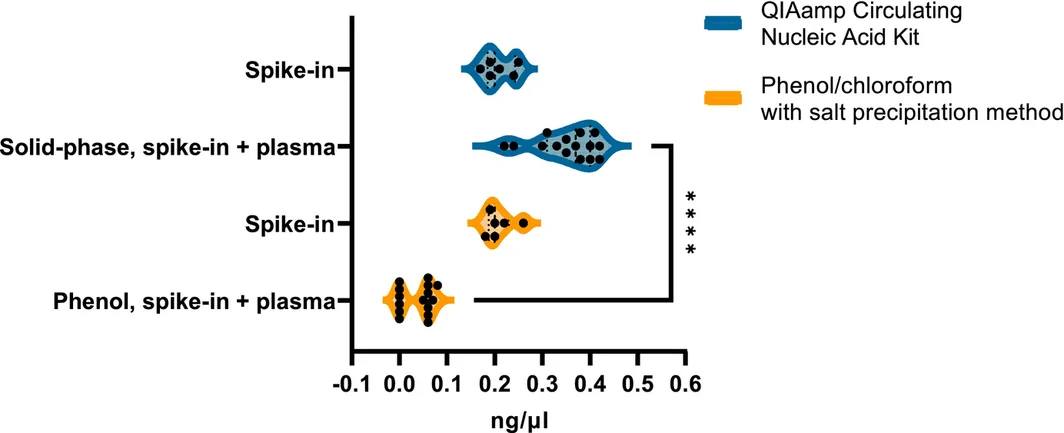
Violin plot presents the total DNA purification yields from plasma for the solid‐phase and phenol/chloroform purification methods. The total DNA was detected using Qubit. Six to fifteen independent measurements were conducted. All data are presented as mean ± standard deviation.

The purification efficiency of the solid‐phase and phenol/chloroform purification was assessed by comparing the total DNA yield, the relative lDNA yields as well as the relative plasmid yields (to baseline controls) before (Bars 6–10) and after exonuclease treatment (Bars 11–15) between each of the two methods (Figures 1 and 2). In terms of overall yield, the solid‐phase method performed better than phenol/chloroform purification (*p* < 0.0001, Figure 3). With regards to relative plasmid purification yields the phenol/chloroform purification outperformed the solid‐phase method for all of the three tested plasmids, p4339 (65.9% vs. 54.6%, *p* ≤ 10^−3^), pBR322 (99.2% vs. 78.8%, *p* ≤ 10^−4^), and pML104 (74.1% vs. 67.0%, *p* ≤ 10^−2^) (Figures 1 and 2, Bars 6–8). Interestingly the phenol/chloroform approach yielded significantly lower yields for each of the plasmids compared with the solid‐phase method following exonuclease treatment; p4339 (18.7% vs. 54.0%, *p* ≤ 10^−4^), pBR322 (26.7% vs. 65.9%, *p* ≤ 10^−4^), and pML104 (15.7% vs. 47.8%, *p* ≤ 10^−4^) (Figures 1 and 2, Bars 11–13), suggesting greater plasmid stability during solid‐phase purification. Finally, the phenol/chloroform approach had a significantly lower lDNA yield compared with the solid‐phase method for both spiked‐in lDNA fragments, 820 bp (55.3% vs 76.5%, *p* ≤ 10^−4^) and 2716 bp (53.1% vs. 96.0%, *p* ≤ 10^−4^) (Bars 9–10) (Figures 1 and 2).

### 3.5 *KRAS* SNP detection and Φ29 amplification

**FIGURE 4:**
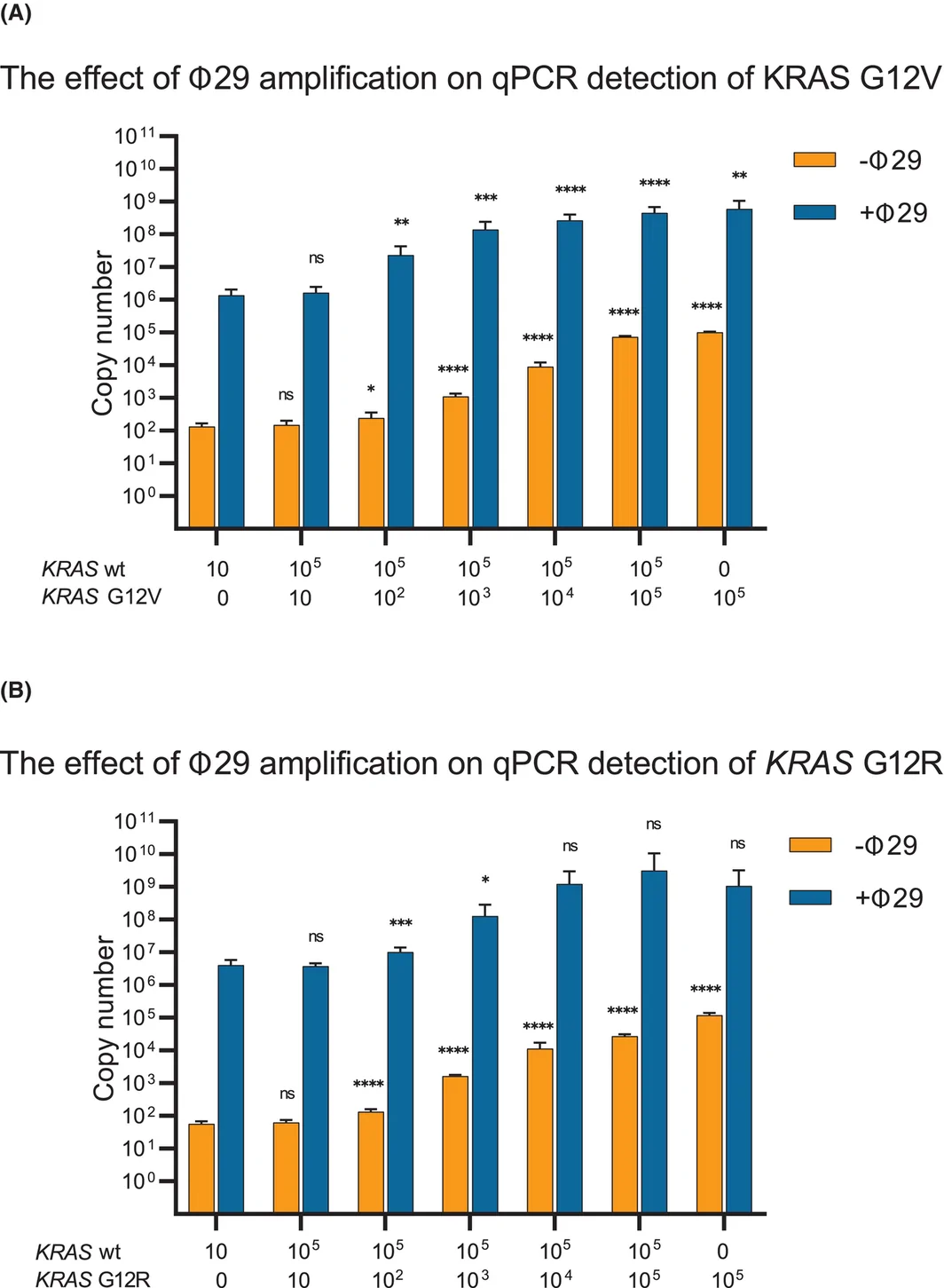
Detection of *KRAS* SNPs in a wt *KRAS* background and the effect of Φ29 amplification on the sensitivity. Three independent purifications were conducted and measured in triplicates by qPCR. (A) detection of the G12V *KRAS* mutant and (B) detection of the G12R *KRAS* mutant. Each bar includes nine data points. Significance was assessed by comparing each detection with the 10^5^ wt background level using a *t*‐test. ns denotes “not significant” (*p* > 0.05), *p ≤ 0.05, **p ≤ 0.01, ***p ≤ 0.001, ****p ≤ 0.0001. All data are presented as mean ± standard deviation.

One possible use of purified eccDNA is its application in the detection of known cancer mutations. We tested qPCR's sensitivity for single nucleotide detection on synthesized circular DNA for two most common cancer mutations in the *RAS* gene family, *KRAS* G12V and *KRAS* G12R, in a mixture of wt *KRAS* containing plasmids (Figure 4). Background signals originating from the SNP allele specific primer's similarity with the wt sequence were taken into account by comparing the mutant detection levels with wt background levels (Figure 4A,B, first bar). We observed a SNP detection sensitivity down to 100 copies of mutant *KRAS* in a background of 10^5^ copies of wt *KRAS* for both mutant alleles when compared with 0 copy mutants, *KRAS* G12V (*p* ≤ 0.05 without amplification and *p* ≤ 0.01 with amplification) (Figure 4A) and *KRAS* G12R (*p* ≤ 0.0001 without amplification and *p* ≤ 0.001 with amplification) (Figure 4B). Rolling circle amplification of the purified DNA prior to qPCR did not increase mutant allele detection sensitivity.

### 3.6 *KRAS* SNP detection and dd‐primer

**FIGURE 5:**
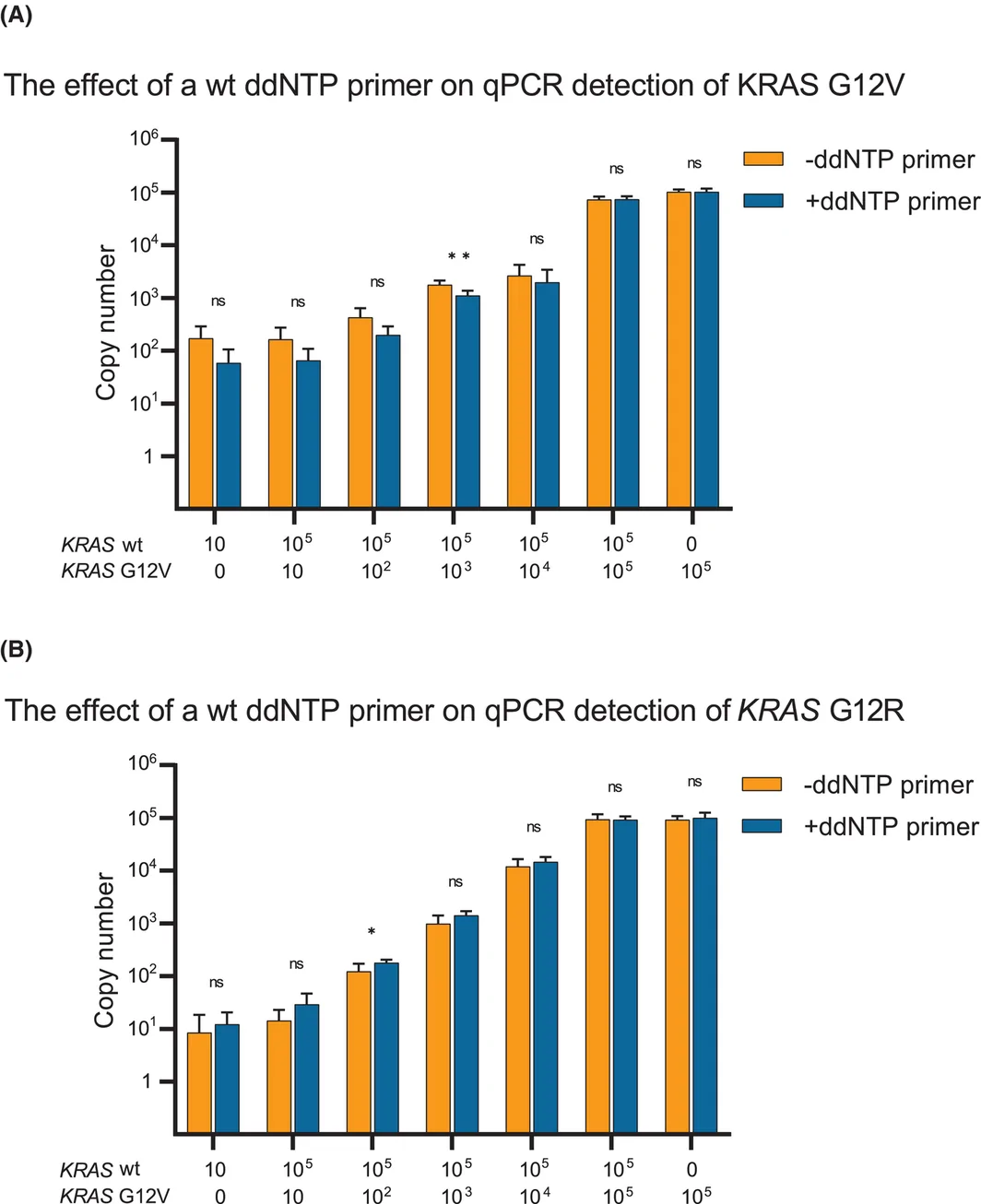
Detection of *KRAS* SNPs in a background of *KRAS* wt with and without dd‐primer *KRAS* wt primer. Three independent purifications were conducted and measured in triplicates by qPCR. (A) shows the G12V *KRAS* mutant and (B) shows the G12R *KRAS* mutant. Each bar represents nine data points. Data were analyzed by *t*‐test. ns denotes “not significant” (*p* > 0.05), *p ≤ 0.05, **p ≤ 0.01, ***p ≤ 0.001, ****p ≤ 0.0001. All data are presented as mean ± standard deviation.

As a potential way of improving sensitivity, we tested the effect of inhibiting wt *KRAS* amplification by including a dd‐primer (3′‐end) wt *KRAS* primer in the qPCR reaction mixture. However, the dd‐primer wt primer inclusion did not significantly increase the assay's sensitivity compared with the dd‐primer‐free detection of the *KRAS* mutants (Figure 5A,B). Neither our attempts at optimizing the annealing temperature nor the amount of dd‐primer relative to the target primers for the G12D mutant were found to improve the detection efficiency (Figures S2 and S3).

### 3.7 Detection of pathogenic SNPs in PDAC plasma samples

To assess the detectable presence of *KRAS* mutations on eccDNA in clinical samples, ~0.5 mL plasma samples from 16 Stage III‐IV PDAC patients and 19 healthy controls (Table 5) were purified using the phenol/chloroform purification method, amplified with Φ29 and sequenced using short‐read, paired‐end sequencing. High‐confidence eccDNA was called using our previously published pipeline and low confidence eccDNA excluded from the analysis.^30^ We identified a mean of 36 eccDNAs pr. sample in the healthy donors and 127 eccDNAs from Stage III‐IV PDAC patients, of which there was one marked outlier with 736 eccDNAs (Table 5). We identified a total of 4223 variants in the 2725 eccDNAs called. Of these, 1410 were associated with genes of which only 20 were annotated in relation to clinical significance in ClinVar. Eighteen of the 20 were identified in PDAC patients, however, all 20 were classified as “benign.” We did not detect any eccDNA originating from the *KRAS* gene. The total DNA content pr. PDAC patient was on average 157,558 bp, corresponding to 0.005% of the haploid human genome. Therefore, even by increasing the numbers of eccDNAs purified several fold by, for example, higher sample volumes and more efficient purification methods, the likelihood of observing a chosen SNP in each sample would be less than 1%.

**TABLE 5:** EccDNA detected in patient plasma.

	Healthy (*n* = 19)	Stage III‐IV PDAC (*n* = 16)
Age (years), mean (95% CI)	63.3 (59.7–66.8)	68.8 (60.4–71.1)
Sex		
Male	6 (31.6%)	8 (50%)
Female	13 (68.4%)	8 (50%)
Plasma volume (mL), mean (95% CI)	0.57 (0.46–0.65)	0.56 (0.46–0.65)
N high confidence eccDNA detected, mean (95% CI)	36.6 (20.6–52.6)	126.8 (33.3–220)
Sum of bp in high confidence eccDNA, mean (95% CI)	24,498 (13,514–35,483)	157,558 (29,807–285,309)
Variants detected in high confidence eccDNA (total)	996	3237
Variants from genes (total)	304	1106
Variants with ClinVar annotation (total)	2	18

Abbreviation: PDAC, pancreatic ductal adenocarcinoma.

## 4 DISCUSSION

With the increasing interest in eccDNA as a biomarker of disease, the need for an established benchmark for eccDNA purification procedures is increasing. In this study, we applied and compared two methods for plasma eccDNA purification, and an approach for the detection of mutant alleles on eccDNA. Our approach included a phenol/chloroform approach with salt precipitation and a column‐based solid‐phase purification method based on the QIAamp commercial kit. We found that the QIAamp‐based purification method combined with exonuclease treatment had the highest recovery rate of spiked‐in circular DNA (>60%), which suggests that the commercially available QIAamp‐based purification approach is a good candidate for a benchmark standard for plasma circular DNA purification assessments. For our experiments, we decided on plasmids ranging in size from 3651 to 11,240 bp to test the purification and stability of potentially clinically relevant circular DNAs, but eccDNA in plasma is primarily found to be present at sizes <1000 bp.^11^, ^12^ Thus our experiment may not fully reflect the biological plasma eccDNA setting.

The most noticeable difference between the yields of the two purification methods was the degree of exonuclease‐susceptible DNA damage sustained by the spiked‐in circular DNA molecules. Although the initial yield of the phenol/chloroform method was generally higher (by 10%–26%) than that achieved using QIAamp (Figures 1 and 2), the amount of linearizing damage suffered by the circular DNA entities during purification (phenol/chloroform 71%–79% DNA damage, QIAamp 1%–29% DNA damage) ensured a higher final circular DNA yield via the QIAamp method (45%–104% greater yield). This variation in final circular DNA yields underlines the importance of testing not only the initial eccDNA purification yield but also the degree of DNA damage suffered during the purification procedure.^34^ Such a comparison is especially important when applying methods such as the phenol/chloroform procedure, which has the potential to cause oxidative damage (8‐hydroxyguanine [8‐OHGua]) to the DNA if the DNA is exposed to oxygen after the phenol has been removed.^35^ Furthermore, each purification procedure is likely to expose the DNA to different amounts of mechanical stress under different environmental conditions, which is known to risk shearing of the DNA.^36^ In our comparative experiment, both the phenol/chloroform and QIAamp protocol applies similar enzymes and centrifugation forces to the DNA, and although there is a greater amount of DNA handling for the phenol/chloroform method, most of it is performed gently and although the plasmids ranged from 4.4 to 11.2 kbp in size, the observed recovery rates were not markedly different (though pML104 had the lowest yield in both methods). It is, therefore, unlikely that mechanical shearing during pipetting would be solely responsible for the large difference observed in circular DNA linearization when our two methods were compared. Furthermore, both purification methods made use of high‐speed centrifugation, pipetting, and, in the case of QIAamp, vortexing, which may all cause mechanical stress and damage to the circular DNA. This could also explain some of the general DNA shearing observed for both methods, which is in line with the largest plasmid (pML104) having the lowest yield after exonuclease treatment for both methods. The DNA is also partially dried and redissolved in both protocols, why variations in dry states are unlikely to be the main cause of damage. This leaves the chemical environment during handling as the most likely explanation for the observed difference in circular DNA linearizing damage.

One thing is to purify the DNA, but to use it in the clinic, it is essential to have a reliable detection method capable of identifying relevant biological targets. We therefore tested the application and sensitivity of qPCR for SNP detection through primer set–specific amplification. As a target, we chose *KRAS* on circular plasmids as a marker, since SNPs in this gene occur in roughly 90% of PDAC patients.^18^, ^19^, ^20^, ^23^ To simulate a biological environment in which the DNA from healthy cells are mixed with the disease targets, we supplemented our solutions with wt *KRAS* containing plasmids.^37^, ^38^ We found that our assay had a sensitivity of ~10^−3^ for cDNA‐located SNPs (100 SNP copies pr. 10^5^ wt). As this may be insufficient for optimal plasma eccDNA biomarker detection, we considered whether the sensitivity was amount or ratio dependent. We therefore tested the detection system following Φ29 (a highly processive DNA polymerase^39^, ^40^) amplification. As Φ29 has a similar amplification rate for circular DNA of the same size,^41^ the amplification is unlikely to disrupt the mutant to wt ratio of our experiments while increasing the material quantity. Since the amplification did not improve the sensitivity of the method, we conclude that the insufficient sensitivity was not due to a quantitative issue but likely related to a methodologic challenge. We therefore sought to reduce the wt signal and improve the signal to noise ratio by using wt *KRAS* primer with dd‐primer at the 3′‐end. ddC is used to block the 3′‐end of oligonucleotides to prevent their extension by a polymerase in a PCR to improve the throughput and accuracy.^42^ Unfortunately, this did not markedly improve the sensitivity of the assay, suggesting that the challenge lies in the detection sensitivity of our qPCR setup rather than the off‐target amplification of the wt target. Having a high sensitivity is essential for a plasma eccDNA‐based assay, as the amount of biological material from a single 1 mL plasma sample only represents 3.64 × 10^−4^ of an average person's ~2.75 L plasma volume, in which the biomarkers of interest are dissolved. As the detection sensitivity of our circular DNA SNP targeting qPCR assay only reached ~10^−3^ against a 10^5^ wt background, we do not find that the presented primer‐based qPCR is an optimal approach for SNP detection. As our findings prove that current PCR tests are insufficient for eccDNA‐based cancer detection and characterizations it may be necessary to rely on sequencing‐based technologies for cancer marker detection. Our profiling of plasma eccDNA from 16 Stage III‐IV PDAC patients and 19 healthy controls for the detection of cancer pathogenic SNP in eccDNA did not reveal any *KRAS*‐derived eccDNAs or eccDNAs with pathogenic variants in the samples from either group. As these samples were purified from ~0.5 mL plasma with the less efficient phenol/chloroform method, we expect that different protocols and larger sample volumes could increase the eccDNA yield substantially. However, we estimate that the likelihood of finding a specific pathogenic SNP in a plasma‐derived sample of eccDNA would still remain low. It would potentially be possible to optimize the sequencing detection of PDAC‐relevant SNPs in plasma, by maintaining both the linear and circular cfDNA molecules and test for additional SNP variants aside from *KRAS*. Alternatively, the characterization, specificity and sensitivity of PDAC detection could possibly be improved using the fragmentomic or methylation profiles of the plasma eccDNA. However, the tools for such an assessment are yet to be developed before such an approach can be generally tested and applied in a clinical setting.

In conclusion, we find that >60% of known circular DNA species can be recovered from plasma using a QIAamp‐based purification approach, which is why we suggest it as a standard benchmark for future plasma eccDNA purification assessments. We also show that qPCR can be used to detect and quantify SNP mutant alleles on circular plasmid DNA with a detection sensitivity of ~10^−3^ for the SNPs (100 SNP copies pr. 10^5^ wt). Finally, we show that detecting a *KRAS* mutation in the plasma‐derived eccDNA from a given patient is unlikely using the current methods. There is therefore, still a need for the development of more sensitive methods for the detection of PDAC using low‐level eccDNA liquid biopsies such as plasma samples.

## AUTHOR CONTRIBUTIONS

**Lasse Bøllehuus Hansen:** Conceptualization (equal); data curation (equal); formal analysis (equal); investigation (supporting); methodology (supporting); project administration (equal); supervision (supporting); validation (supporting); visualization (supporting); writing – original draft (lead); writing – review and editing (lead). **Sandra Fugl Jakobsen:** Data curation (lead); formal analysis (lead); investigation (lead); methodology (equal); validation (lead); visualization (lead); writing – original draft (lead); writing – review and editing (supporting). **Egija Zole:** Formal analysis (equal); supervision (supporting); writing – original draft (lead); writing – review and editing (equal). **Julie Boertmann Noer:** Methodology (equal); supervision (equal); writing – review and editing (supporting). **Li Tai Fang:** Writing – review and editing (equal). **Sefa Alizadeh:** Formal analysis (supporting); methodology (supporting); supervision (supporting). **Julia Sidenius Johansen:** Writing – review and editing (equal). **Marghoob Mohiyuddin:** Writing – review and editing (equal). **Birgitte Regenberg:** Conceptualization (lead); data curation (equal); formal analysis (equal); funding acquisition (lead); investigation (supporting); methodology (supporting); project administration (equal); resources (lead); supervision (equal); validation (supporting); visualization (supporting); writing – original draft (supporting); writing – review and editing (equal).

## FUNDING INFORMATION

LBH, SFJ, JBN, SA, BR, and JSH were funded by the Innovation Fund Denmark (CARE DNA, grant number 8088‐00049B). BR and EZ were also funded by Horizon 2020 (CIRCULAR VISION, grant number 899417). LBH, JBN, BR and JSH were also funded by Sygesikring Danmark (CIRCPAC, grant number 2021‐0321).

## CONFLICT OF INTEREST STATEMENT

LT and MM are employees of Roche. LBH, SFJ, JBN, SA, BR, and JSH declare that they have no known competing financial interests or personal relationships that could have appeared to influence the work reported in this paper.

## Supporting information


Data S1:

## Data Availability

Data regarding plasma eccDNA from clinical patients (BIOPAC) is available from the corresponding author upon reasonable request.
