# Supplementary material for: Methods for the purification and detection of single nucleotide KRAS mutations on extrachromosomal circular DNA in human plasma
Source: Cancer Med. 2023 Aug 21;12(17):17679–91. doi: 10.1002/cam4.6385 (PMC10523981; doi:10.1002/cam4.6385)
Supplement: Supplementary file 1 — Data S1: [file CAM4-12-17679-s001.docx]

**Supplementary Figure 1**

**Plasmid p4339**


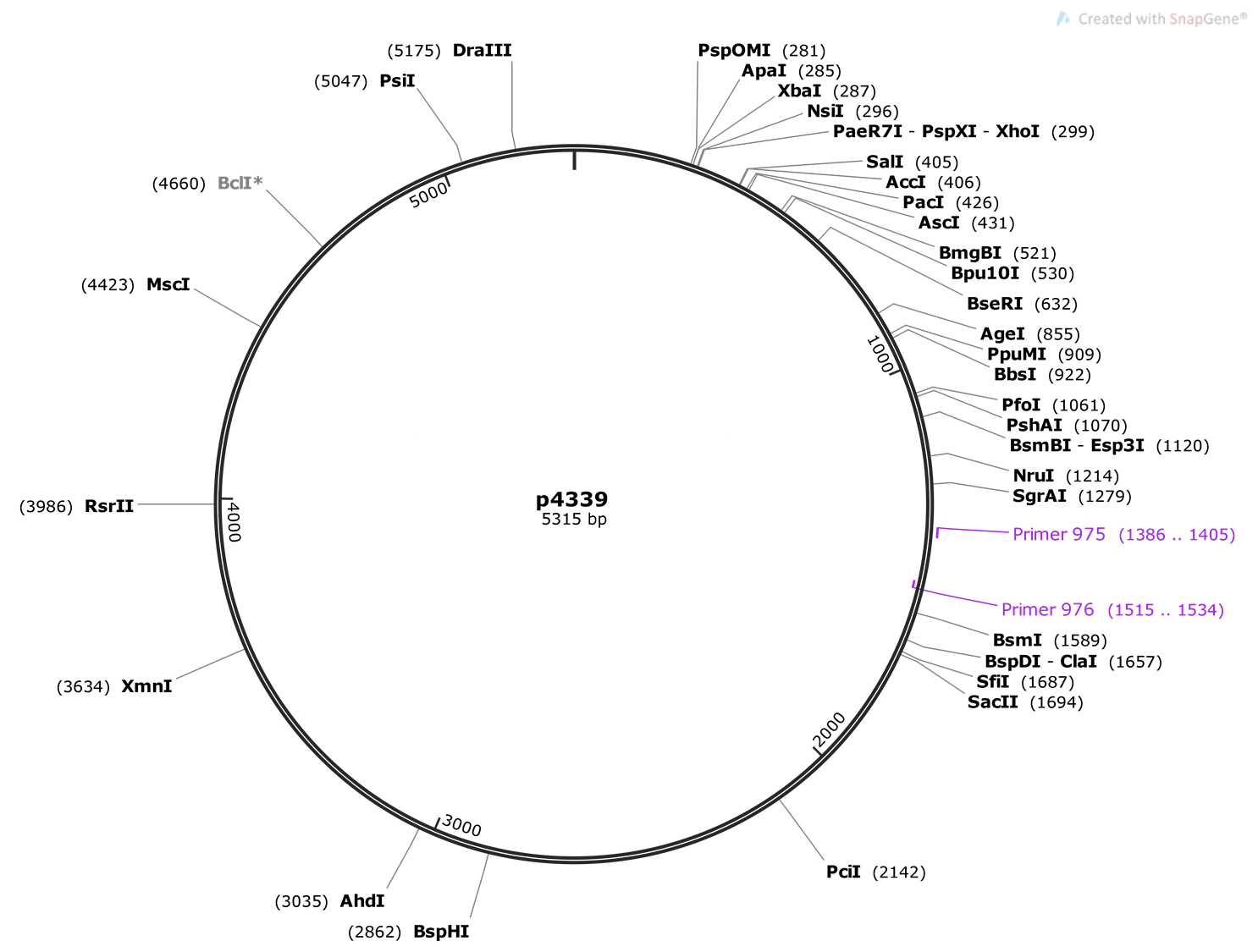


Plasmid sequence:

GGAGCGGGCGCTAGGGCGCTGGCAAGTGTAGCGGTCACGCTGCGCGTAACCACCACACCCGCCGCGCTTAATGCGCCGCTACAGGGCGCGTCCATTCGCCATTCAGGCTGCGCAACTGTTGGGAAGGGCGATCGGTGCGGGCCTCTTCGCTATTACGCCAGCTGGCGAAAGGGGGATGTGCTGCAAGGCGATTAAGTTGGGTAACGCCAGGGTTTTCCCAGTCACGACGTTGTAAAACGACGGCCAGTGAATTGTAATACGACTCACTATAGGGCGAATTGGGCCCTCTAGATGCATGCTCGAGCGGCCGCCAGTGTGATGGATATCTGCAGAATTCGCCCTTCACATACGATTTAGGTGACACTATAGAACGCGGCCGCCAGCTGAAGCTTCGTACGCTGCAGGTCGACGGATCCCCGGGTTAATTAAGGCGCGCCAGATCTGTTTAGCTTGCCTCGTCCCCGCCGGGTCACCCGGCCAGCGACATGGAGGCCCAGAATACCCTCCTTGACAGTCTTGACGTGCGCAGCTCAGGGGCATGATGTGACTGTCGCCCGTACATTTAGCCCATACATCCCCATGTATAATCATTTGCATCCATACATTTTGATGGCCGCACGGCGCGAAGCAAAAATTACGGCTCCTCGCTGCAGACCTGCGAGCAGGGAAACGCTCCCCTCACAGACGCGTTGAATTGTCCCCACGCCGCGCCCCTGTAGAGAAATATAAAAGGTTAGGATTTGCCACTGAGGTTCTTCTTTCATATACTTCCTTTTAAAATCTTGCTAGGATACAGTTCTCACATCACATCCGAACATAAACAACCATGGGTACCACTCTTGACGACACGGCTTACCGGTACCGCACCAGTGTCCCGGGGGACGCCGAGGCCATCGAGGCACTGGATGGGTCCTTCACCACCGACACCGTCTTCCGCGTCACCGCCACCGGGGACGGCTTCACCCTGCGGGAGGTGCCGGTGGACCCGCCCCTGACCAAGGTGTTCCCCGACGACGAATCGGACGACGAATCGGACGACGGGGAGGACGGCGACCCGGACTCCCGGACGTTCGTCGCGTACGGGGACGACGGCGACCTGGCGGGCTTCGTGGTCGTCTCGTACTCCGGCTGGAACCGCCGGCTGACCGTCGAGGACATCGAGGTCGCCCCGGAGCACCGGGGGCACGGGGTCGGGCGCGCGTTGATGGGGCTCGCGACGGAGTTCGCCCGCGAGCGGGGCGCCGGGCACCTCTGGCTGGAGGTCACCAACGTCAACGCACCGGCGATCCACGCGTACCGGCGGATGGGGTTCACCCTCTGCGGCCTGGACACCGCCCTGTACGACGGCACCGCCTCGGACGGCGAGCAGGCGCTCTACATGAGCATGCCCTGCCCCTAATCAGTACTGACAATAAAAAGATTCTTGTTTTCAAGAACTTGTCATTTGTATAGTTTTTTTATATTGTAGTTGTTCTATTTTAATCAAATGTTAGCGTGATTTATATTTTTTTTCGCCTCGACATCATCTGCCCAGATGCGAAGTTAAGTGCGCAGAAAGTAATATCATGCGTCAATCGTATGTGAATGCTGGTCGCTATACTGCTGTCGATTCGATACTAACGCCGCCATCCAGTGTCGAAAACGAGCTCGAATTCATCGATGATATCAGATCCACTAGTGGCCTATGCGGCCGCGGATCTGCCGGTCTCCCTATAGTGAGTCGTATTAAGGGCGAATTCCAGCACACTGGCGGCCGTTACTAGTGGATCCGAGCTCGGTACCAAGCTTGGCGTAATCATGGTCATAGCTGTTTCCTGTGTGAAATTGTTATCCGCTCACAATTCCACACAACATACGAGCCGGAAGCATAAAGTGTAAAGCCTGGGGTGCCTAATGAGTGAGCTAACTCACATTAATTGCGTTGCGCTCACTGCCCGCTTTCCAGTCGGGAAACCTGTCGTGCCAGCTGCATTAATGAATCGGCCAACGCGCGGGGAGAGGCGGTTTGCGTATTGGGCGCTCTTCCGCTTCCTCGCTCACTGACTCGCTGCGCTCGGTCGTTCGGCTGCGGCGAGCGGTATCAGCTCACTCAAAGGCGGTAATACGGTTATCCACAGAATCAGGGGATAACGCAGGAAAGAACATGTGAGCAAAAGGCCAGCAAAAGGCCAGGAACCGTAAAAAGGCCGCGTTGCTGGCGTTTTTCCATAGGCTCCGCCCCCCTGACGAGCATCACAAAAATCGACGCTCAAGTCAGAGGTGGCGAAACCCGACAGGACTATAAAGATACCAGGCGTTTCCCCCTGGAAGCTCCCTCGTGCGCTCTCCTGTTCCGACCCTGCCGCTTACCGGATACCTGTCCGCCTTTCTCCCTTCGGGAAGCGTGGCGCTTTCTCATAGCTCACGCTGTAGGTATCTCAGTTCGGTGTAGGTCGTTCGCTCCAAGCTGGGCTGTGTGCACGAACCCCCCGTTCAGCCCGACCGCTGCGCCTTATCCGGTAACTATCGTCTTGAGTCCAACCCGGTAAGACACGACTTATCGCCACTGGCAGCAGCCACTGGTAACAGGATTAGCAGAGCGAGGTATGTAGGCGGTGCTACAGAGTTCTTGAAGTGGTGGCCTAACTACGGCTACACTAGAAGAACAGTATTTGGTATCTGCGCTCTGCTGAAGCCAGTTACCTTCGGAAAAAGAGTTGGTAGCTCTTGATCCGGCAAACAAACCACCGCTGGTAGCGGTGGTTTTTTTGTTTGCAAGCAGCAGATTACGCGCAGAAAAAAAGGATCTCAAGAAGATCCTTTGATCTTTTCTACGGGGTCTGACGCTCAGTGGAACGAAAACTCACGTTAAGGGATTTTGGTCATGAGATTATCAAAAAGGATCTTCACCTAGATCCTTTTAAATTAAAAATGAAGTTTTAAATCAATCTAAAGTATATATGAGTAAACTTGGTCTGACAGTTACCAATGCTTAATCAGTGAGGCACCTATCTCAGCGATCTGTCTATTTCGTTCATCCATAGTTGCCTGACTCCCCGTCGTGTAGATAACTACGATACGGGAGGGCTTACCATCTGGCCCCAGTGCTGCAATGATACCGCGAGACCCACGCTCACCGGCTCCAGATTTATCAGCAATAAACCAGCCAGCCGGAAGGGCCGAGCGCAGAAGTGGTCCTGCAACTTTATCCGCCTCCATCCAGTCTATTAATTGTTGCCGGGAAGCTAGAGTAAGTAGTTCGCCAGTTAATAGTTTGCGCAACGTTGTTGCCATTGCTACAGGCATCGTGGTGTCACGCTCGTCGTTTGGTATGGCTTCATTCAGCTCCGGTTCCCAACGATCAAGGCGAGTTACATGATCCCCCATGTTGTGCAAAAAAGCGGTTAGCTCCTTCGGTCCTCCGATCGTTGTCAGAAGTAAGTTGGCCGCAGTGTTATCACTCATGGTTATGGCAGCACTGCATAATTCTCTTACTGTCATGCCATCCGTAAGATGCTTTTCTGTGACTGGTGAGTACTCAACCAAGTCATTCTGAGAATAGTGTATGCGGCGACCGAGTTGCTCTTGCCCGGCGTCAATACGGGATAATACCGCGCCACATAGCAGAACTTTAAAAGTGCTCATCATTGGAAAACGTTCTTCGGGGCGAAAACTCTCAAGGATCTTACCGCTGTTGAGATCCAGTTCGATGTAACCCACTCGTGCACCCAACTGATCTTCAGCATCTTTTACTTTCACCAGCGTTTCTGGGTGAGCAAAAACAGGAAGGCAAAATGCCGCAAAAAAGGGAATAAGGGCGACACGGAAATGTTGAATACTCATACTCTTCCTTTTTCAATTCAGAAGAACTCGTCAAGAAGGCGATAGAAGGCGATGCGCTGCGAATCGGGAGCGGCGATACCGTAAAGCACGAGGAAGCGGTCAGCCCATTCGCCGCCAAGCTCTTCAGCAATATCACGGGTAGCCAACGCTATGTCCTGATAGCGGTCCGCCACACCCAGCCGGCCACAGTCGATGAATCCAGAAAAGCGGCCATTTTCCACCATGATATTCGGCAAGCAGGCATCGCCATGGGTCACGACGAGATCCTCGCCGTCGGGCATGCGCGCCTTGAGCCTGGCGAACAGTTCGGCTGGCGCGAGCCCCTGATGCTCTTCGTCCAGATCATCCTGATCGACAAGACCGGCTTCCATCCGAGTACGTGCTCGCTCGATGCGATGTTTCGCTTGGTGGTCGAATGGGCAGGTAGCCGGATCAAGCGTATGCAGCCGCCGCATTGCATCAGCCATGATGGATACTTTCTCGGCAGGAGCAAGGTGGGATGACAGGAGATCCTGCCCCGGCACTTCGCCCAATAGCAGCCAGTCCCTTCCCGCTTCAGTGACAACGTCGAGCACAGCTGCGCAAGGAACGCCCGTCGTGGCCAGCCACGATAGCCGCGCTGCCTCGTCCTGCAGTTCATTCAGGGCACCGGACAGGTCGGTCTTGACAAAAAGAACCGGGCGCCCCTGCGCTGACAGCCGGAACACGGCGGCATCAGAGCAGCCGATTGTCTGTTGTGCCCAGTCATAGCCGAATAGCCTCTCCACCCAAGCGGCCGGAGAACCTGCGTGCAATCCATCTTGTTCAATCATGCGAAACGATCCTCATCCTGTCTCTTGATCAGATCTTGATCCCCTGCGCCATCAGATCCTTGGCGGCAAGAAAGCCATCCAGTTTACTTTGCAGGGCTTCCCAACCTTACCAGAGGGCGCCCCAGCTGGCAATTCCGGTTCGCTTGCTGTCCATAAAACCGCCCAGTCTAGCTATCGCCATGTAAGCCCACTGCAAGCTACCTGCTTTCTCTTTGCGCTTGCGTTTTCCCTTGTCCAGATAGCCCAGTAGCTGACATTCATCCGGGGTCAGCACCGTTTCTGCGGACTGGCTTTCTACGTGTTCCGCTTCCTTTAGCAGCCCTTGCGCCCTGAATTTTGTTAAAATTCGCGTTAAATTTTTGTTAAATCAGCTCATTTTTTAACCAATAGGCCGAAATCGGCAAAATCCCTTATAAATCAAAAGAATAGACCGAGATAGGGTTGAGTGTTGTTCCAGTTTGGAACAAGAGTCCACTATTAAAGAACGTGGACTCCAACGTCAAAGGGCGAAAAACCGTCTATCAGGGCGATGGCCCACTACGTGAACCATCACCCTAATCAAGTTTTTTGGGGTCGAGGTGCCGTAAAGCACTAAATCGGAACCCTAAAGGGAGCCCCCGATTTAGAGCTTGACGGGGAAAGCCGGCGAACGTGGCGAGAAAGGAAGGGAAGAAAGCGAAA

**Plasmid pML104**


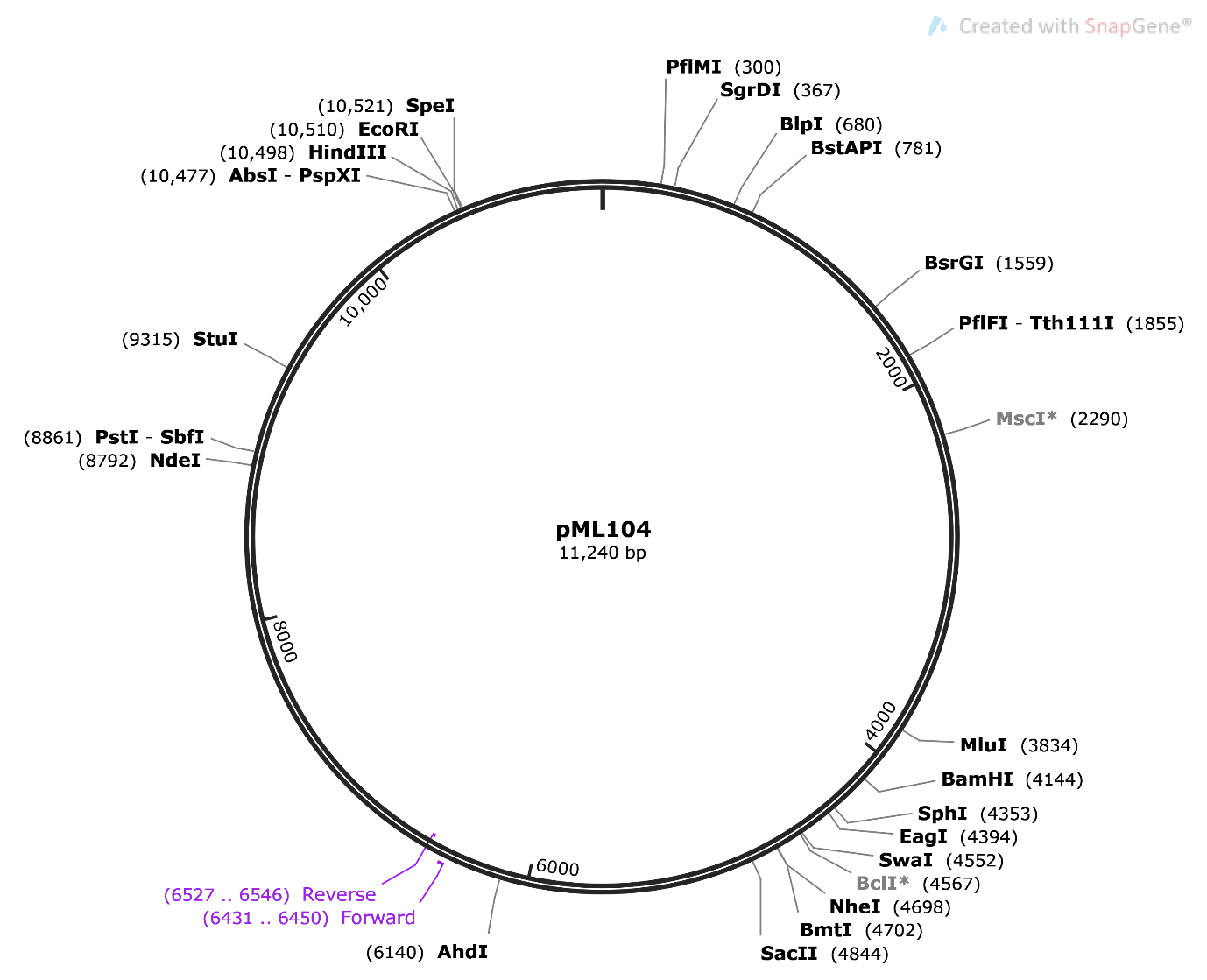


Plasmid sequence:

ATGGACAAGAAGTATTCTATCGGACTGGACATCGGGACTAATAGCGTCGGGTGGGCCGTCATCACTGACGAGTACAAGGTGCCCTCTAAGAAGTTCAAGGTGCTCGGGAACACCGACCGGCATTCCATCAAGAAAAATCTGATCGGAGCTCTCCTCTTTGATTCAGGGGAGACCGCTGAAGCAACCCGCCTCAAGCGGACTGCTAGACGGCGGTACACCAGGAGGAAGAACCGGATTTGTTACCTTCAAGAGATATTCTCCAACGAAATGGCAAAGGTCGACGACAGCTTCTTCCATAGGCTGGAAGAATCATTCCTCGTGGAAGAGGATAAGAAGCATGAACGGCATCCCATCTTCGGTAATATCGTCGACGAGGTGGCCTATCACGAGAAATACCCAACCATCTACCATCTTCGCAAAAAGCTGGTGGACTCAACCGACAAGGCAGACCTCCGGCTTATCTACCTGGCCCTGGCCCACATGATTAAGTTCAGAGGCCACTTCCTGATCGAGGGCGACCTCAATCCTGACAATAGCGATGTGGATAAACTGTTCATCCAGCTGGTGCAGACTTACAACCAGCTCTTTGAAGAGAACCCCATCAATGCAAGCGGAGTCGATGCCAAGGCCATTCTGTCAGCCCGGCTGTCAAAGAGCCGCAGACTTGAGAATCTTATCGCTCAGCTGCCGGGTGAAAAGAAAAATGGACTGTTCGGGAACCTGATTGCTCTTTCACTTGGGCTGACTCCCAATTTCAAGTCTAATTTCGACCTGGCAGAGGATGCCAAGCTGCAACTGTCCAAGGACACCTATGATGACGATCTCGACAACCTCCTGGCCCAGATCGGTGACCAATACGCCGACCTTTTCCTTGCTGCTAAGAATCTTTCTGACGCCATCCTGCTGTCTGACATTCTCCGCGTGAACACTGAAATCACCAAGGCCCCTCTTTCAGCTTCAATGATTAAGCGGTATGATGAGCACCACCAGGACCTGACCCTGCTTAAGGCACTCGTCCGGCAGCAGCTTCCGGAGAAGTACAAGGAAATCTTCTTTGACCAGTCAAAGAATGGATACGCCGGCTACATCGACGGAGGTGCCTCCCAAGAGGAATTTTATAAGTTTATCAAACCTATCCTTGAGAAGATGGACGGCACCGAAGAGCTCCTCGTGAAACTGAATCGGGAGGATCTGCTGCGGAAGCAGCGCACTTTCGACAATGGGAGCATTCCCCACCAGATCCATCTTGGGGAGCTTCACGCCATCCTTCGGCGCCAAGAGGACTTCTACCCCTTTCTTAAGGACAACAGGGAGAAGATTGAGAAAATTCTCACTTTCCGCATCCCCTACTACGTGGGACCCCTCGCCAGAGGAAATAGCCGGTTTGCTTGGATGACCAGAAAGTCAGAAGAAACTATCACTCCCTGGAACTTCGAAGAGGTGGTGGACAAGGGAGCCAGCGCTCAGTCATTCATCGAACGGATGACTAACTTCGATAAGAACCTCCCCAATGAGAAGGTCCTGCCGAAACATTCCCTGCTCTACGAGTACTTTACCGTGTACAACGAGCTGACCAAGGTGAAATATGTCACCGAAGGGATGAGGAAGCCCGCATTCCTGTCAGGCGAACAAAAGAAGGCAATTGTGGACCTTCTGTTCAAGACCAATAGAAAGGTGACCGTGAAGCAGCTGAAGGAGGACTATTTCAAGAAAATTGAATGCTTCGACTCTGTGGAGATTAGCGGGGTCGAAGATCGGTTCAACGCAAGCCTGGGTACCTACCATGATCTGCTTAAGATCATCAAGGACAAGGATTTTCTGGACAATGAGGAGAAAGAGGACATCCTTGAGGACATTGTCCTGACTCTCACTCTGTTCGAGGACCGGGAAATGATCGAGGAGAGGCTTAAGACCTACGCCCATCTGTTCGACGATAAAGTGATGAAGCAACTTAAACGGAGAAGATATACCGGATGGGGACGCCTTAGCCGCAAACTCATCAACGGAATCCGGGACAAACAGAGCGGAAAGACCATTCTTGATTTCCTTAAGAGCGACGGATTCGCTAATCGCAACTTCATGCAACTTATCCATGATGATTCCCTGACCTTTAAGGAGGACATCCAGAAGGCCCAAGTGTCTGGACAAGGTGACTCACTGCACGAGCATATCGCAAATCTGGCTGGTTCACCCGCTATTAAGAAGGGTATTCTCCAGACCGTGAAAGTCGTGGACGAGCTGGTCAAGGTGATGGGTCGCCATAAACCAGAGAACATTGTCATCGAGATGGCCAGGGAAAACCAGACTACCCAGAAGGGACAGAAGAACAGCAGGGAGCGGATGAAAAGAATTGAGGAAGGGATTAAGGAGCTCGGGTCACAGATCCTTAAAGAGCACCCGGTGGAAAACACCCAGCTTCAGAATGAGAAGCTCTATCTGTACTACCTTCAAAATGGACGCGATATGTATGTGGACCAAGAGCTTGATATCAACAGGCTCTCAGACTACGACGTGGACCACATCGTCCCTCAGAGCTTCCTCAAAGACGACTCAATTGACAATAAGGTGCTGACTCGCTCAGACAAGAACCGGGGAAAGTCAGATAACGTGCCCTCAGAGGAAGTCGTGAAAAAGATGAAGAACTATTGGCGCCAGCTTCTGAACGCAAAGCTAATCACTCAGCGGAAGTTCGACAATCTCACTAAGGCTGAGAGGGGCGGACTGAGCGAACTGGACAAAGCAGGATTCATTAAACGGCAACTTGTGGAGACTCGGCAGATTACTAAACATGTAGCCCAAATCCTTGACTCACGCATGAATACCAAGTACGACGAAAACGACAAACTTATCCGCGAGGTGAAGGTGATTACCCTGAAGTCCAAGCTGGTCAGCGATTTCAGAAAGGACTTTCAATTCTACAAAGTGCGGGAGATCAATAACTATCATCATGCTCATGACGCATATCTGAATGCCGTGGTGGGAACCGCCCTAATCAAGAAGTACCCAAAGCTGGAAAGCGAGTTCGTGTACGGAGACTACAAGGTCTACGACGTGCGCAAGATGATTGCCAAATCTGAGCAGGAGATCGGAAAGGCCACCGCAAAGTACTTCTTCTACAGCAACATCATGAATTTCTTCAAGACCGAAATCACCCTTGCAAACGGTGAGATCCGGAAGAGGCCGCTCATCGAGACTAATGGGGAGACTGGCGAAATCGTGTGGGACAAGGGCAGAGATTTCGCTACCGTGCGCAAAGTGCTTTCTATGCCTCAAGTGAACATCGTGAAGAAAACCGAGGTGCAAACCGGAGGCTTTTCTAAGGAATCAATCCTCCCCAAGCGCAACTCCGACAAGCTCATTGCAAGGAAGAAGGATTGGGACCCTAAGAAGTACGGCGGATTCGATTCACCAACTGTGGCTTATTCTGTCCTGGTCGTGGCTAAGGTGGAAAAAGGAAAGTCTAAGAAGCTCAAGAGCGTGAAGGAACTGCTGGGTATCACCATTATGGAGCGCAGCTCCTTCGAGAAGAACCCAATTGACTTTCTCGAAGCCAAAGGTTACAAGGAAGTCAAGAAGGACCTTATCATCAAGCTCCCAAAGTATAGCCTGTTCGAACTGGAGAATGGGCGGAAGCGGATGCTCGCCTCCGCTGGCGAACTTCAGAAGGGTAATGAGCTGGCTCTCCCCTCCAAGTACGTGAATTTCCTCTACCTTGCAAGCCATTACGAGAAGCTGAAGGGGAGCCCCGAGGACAACGAGCAAAAGCAACTGTTTGTGGAGCAGCATAAGCATTATCTGGACGAGATCATTGAGCAGATTTCCGAGTTTTCTAAACGCGTCATTCTCGCTGATGCCAACCTCGATAAAGTCCTTAGCGCATACAATAAGCACAGAGACAAACCAATTCGGGAGCAGGCTGAGAATATCATCCACCTGTTCACCCTCACCAATCTTGGTGCCCCTGCCGCATTCAAGTACTTCGACACCACCATCGACCGGAAACGCTATACCTCCACCAAAGAAGTGCTGGACGCCACCCTCATCCACCAGAGCATCACCGGACTTTACGAAACTCGGATTGACCTCTCACAGCTCGGAGGGGATGAGGGAGCTCCCAAGAAAAAGCGCAAGGTAGGTAGTTCCGGATCCTAGACTCTCGAGGCGAATTTCTTATGATTTATGATTTTTATTATTAAATAAGTTATAAAAAAAATAAGTGTATACAAATTTTAAAGTGACTCTTAGGTTTTAAAACGAAAATTCTTATTCTTGAGTAACTCTTTCCTGTAGGTCAGGTTGCTTTCTCAGGTATAGCATGAGGTCGCTCTTATTGACCACACCTCTACCGGCATGCCGAGCAAATGCCTGCAAATCGCTCCCCATTTCTCTAGAGCGGCCGTGGTATCGTTTAGATTGGCAATTACAGTGTCTTAGCTCACATGCTTATAACTAATTACATGACTCGAAGACATAAAAAACAAAAAAAGCACCACCGACTCGGTGCCACTTTTTCAAGTTGATAACGGACTAGCCTTATTTTAACTTGCTATTTAAATGCATCGATTCTGATCATTTATCTTTCACTGCGGAGAAGTTTCGAACGCCGAAACATGCGCACCAACTTTCACTTCTACAGCGTTTGACCAAAATCTTTTGAACAGAACATTGTAGGGTGTGAAAAAATGCGCACCTTTACCGCTAGCCCAAGAGGGCACTACAAAATCTAGAGTTGTACTTCAAACGTACATGTAATCACCTTGTATATACTCGAAAGAAAACATCAAGTTTCTGTATAAATATGAGTGAAAGCATAATCATACATTATCTTTTCAAAGATTGTCCGCGGTGGAGCTCCAGCTTTTGTTCCCTTTAGTGAGGGTTAATTGCGCGCTTGGCGTAATCATGGTCATAGCTGTTTCCTGTGTGAAATTGTTATCCGCTCACAATTCCACACAACATACGAGCCGGAAGCATAAAGTGTAAAGCCTGGGGTGCCTAATGAGTGAGCTAACTCACATTAATTGCGTTGCGCTCACTGCCCGCTTTCCAGTCGGGAAACCTGTCGTGCCAGCTGCATTAATGAATCGGCCAACGCGCGGGGAGAGGCGGTTTGCGTATTGGGCGCTCTTCCGCTTCCTCGCTCACTGACTCGCTGCGCTCGGTCGTTCGGCTGCGGCGAGCGGTATCAGCTCACTCAAAGGCGGTAATACGGTTATCCACAGAATCAGGGGATAACGCAGGAAAGAACATGTGAGCAAAAGGCCAGCAAAAGGCCAGGAACCGTAAAAAGGCCGCGTTGCTGGCGTTTTTCCATAGGCTCCGCCCCCCTGACGAGCATCACAAAAATCGACGCTCAAGTCAGAGGTGGCGAAACCCGACAGGACTATAAAGATACCAGGCGTTTCCCCCTGGAAGCTCCCTCGTGCGCTCTCCTGTTCCGACCCTGCCGCTTACCGGATACCTGTCCGCCTTTCTCCCTTCGGGAAGCGTGGCGCTTTCTCATAGCTCACGCTGTAGGTATCTCAGTTCGGTGTAGGTCGTTCGCTCCAAGCTGGGCTGTGTGCACGAACCCCCCGTTCAGCCCGACCGCTGCGCCTTATCCGGTAACTATCGTCTTGAGTCCAACCCGGTAAGACACGACTTATCGCCACTGGCAGCAGCCACTGGTAACAGGATTAGCAGAGCGAGGTATGTAGGCGGTGCTACAGAGTTCTTGAAGTGGTGGCCTAACTACGGCTACACTAGAAGAACAGTATTTGGTATCTGCGCTCTGCTGAAGCCAGTTACCTTCGGAAAAAGAGTTGGTAGCTCTTGATCCGGCAAACAAACCACCGCTGGTAGCGGTGGTTTTTTTGTTTGCAAGCAGCAGATTACGCGCAGAAAAAAAGGATCTCAAGAAGATCCTTTGATCTTTTCTACGGGGTCTGACGCTCAGTGGAACGAAAACTCACGTTAAGGGATTTTGGTCATGAGATTATCAAAAAGGATCTTCACCTAGATCCTTTTAAATTAAAAATGAAGTTTTAAATCAATCTAAAGTATATATGAGTAAACTTGGTCTGACAGTTACCAATGCTTAATCAGTGAGGCACCTATCTCAGCGATCTGTCTATTTCGTTCATCCATAGTTGCCTGACTCCCCGTCGTGTAGATAACTACGATACGGGAGGGCTTACCATCTGGCCCCAGTGCTGCAATGATACCGCGAGACCCACGCTCACCGGCTCCAGATTTATCAGCAATAAACCAGCCAGCCGGAAGGGCCGAGCGCAGAAGTGGTCCTGCAACTTTATCCGCCTCCATCCAGTCTATTAATTGTTGCCGGGAAGCTAGAGTAAGTAGTTCGCCAGTTAATAGTTTGCGCAACGTTGTTGCCATTGCTACAGGCATCGTGGTGTCACGCTCGTCGTTTGGTATGGCTTCATTCAGCTCCGGTTCCCAACGATCAAGGCGAGTTACATGATCCCCCATGTTGTGCAAAAAAGCGGTTAGCTCCTTCGGTCCTCCGATCGTTGTCAGAAGTAAGTTGGCCGCAGTGTTATCACTCATGGTTATGGCAGCACTGCATAATTCTCTTACTGTCATGCCATCCGTAAGATGCTTTTCTGTGACTGGTGAGTACTCAACCAAGTCATTCTGAGAATAGTGTATGCGGCGACCGAGTTGCTCTTGCCCGGCGTCAATACGGGATAATACCGCGCCACATAGCAGAACTTTAAAAGTGCTCATCATTGGAAAACGTTCTTCGGGGCGAAAACTCTCAAGGATCTTACCGCTGTTGAGATCCAGTTCGATGTAACCCACTCGTGCACCCAACTGATCTTCAGCATCTTTTACTTTCACCAGCGTTTCTGGGTGAGCAAAAACAGGAAGGCAAAATGCCGCAAAAAAGGGAATAAGGGCGACACGGAAATGTTGAATACTCATACTCTTCCTTTTTCAATATTATTGAAGCATTTATCAGGGTTATTGTCTCATGAGCGGATACATATTTGAATGTATTTAGAAAAATAAACAAATAGGGGTTCCGCGCACATTTCCCCGAAAAGTGCCACCTGAACGAAGCATCTGTGCTTCATTTTGTAGAACAAAAATGCAACGCGAGAGCGCTAATTTTTCAAACAAAGAATCTGAGCTGCATTTTTACAGAACAGAAATGCAACGCGAAAGCGCTATTTTACCAACGAAGAATCTGTGCTTCATTTTTGTAAAACAAAAATGCAACGCGAGAGCGCTAATTTTTCAAACAAAGAATCTGAGCTGCATTTTTACAGAACAGAAATGCAACGCGAGAGCGCTATTTTACCAACAAAGAATCTATACTTCTTTTTTGTTCTACAAAAATGCATCCCGAGAGCGCTATTTTTCTAACAAAGCATCTTAGATTACTTTTTTTCTCCTTTGTGCGCTCTATAATGCAGTCTCTTGATAACTTTTTGCACTGTAGGTCCGTTAAGGTTAGAAGAAGGCTACTTTGGTGTCTATTTTCTCTTCCATAAAAAAAGCCTGACTCCACTTCCCGCGTTTACTGATTACTAGCGAAGCTGCGGGTGCATTTTTTCAAGATAAAGGCATCCCCGATTATATTCTATACCGATGTGGATTGCGCATACTTTGTGAACAGAAAGTGATAGCGTTGATGATTCTTCATTGGTCAGAAAATTATGAACGGTTTCTTCTATTTTGTCTCTATATACTACGTATAGGAAATGTTTACATTTTCGTATTGTTTTCGATTCACTCTATGAATAGTTCTTACTACAATTTTTTTGTCTAAAGAGTAATACTAGAGATAAACATAAAAAATGTAGAGGTCGAGTTTAGATGCAAGTTCAAGGAGCGAAAGGTGGATGGGTAGGTTATATAGGGATATAGCACAGAGATATATAGCAAAGAGATACTTTTGAGCAATGTTTGTGGAAGCGGTATTCGCAATATTTTAGTAGCTCGTTACAGTCCGGTGCGTTTTTGGTTTTTTGAAAGTGCGTCTTCAGAGCGCTTTTGGTTTTCAAAAGCGCTCTGAAGTTCCTATACTTTCTAGAGAATAGGAACTTCGGAATAGGAACTTCAAAGCGTTTCCGAAAACGAGCGCTTCCGAAAATGCAACGCGAGCTGCGCACATACAGCTCACTGTTCACGTCGCACCTATATCTGCGTGTTGCCTGTATATATATATACATGAGAAGAACGGCATAGTGCGTGTTTATGCTTAAATGCGTACTTATATGCGTCTATTTATGTAGGATGAAAGGTAGTCTAGTACCTCCTGTGATATTATCCCATTCCATGCGGGGTATCGTATGCTTCCTTCAGCACTACCCTTTAGCTGTTCTATATGCTGCCACTCCTCAATTGGATTAGTCTCATCCTTCAATGCTATCATTTCCTTTGATATTGGATCATACTAAGAAACCATTATTATCATGACATTAACCTATAAAAATAGGCGTATCACGAGGCCCTTTCGTCTCGCGCGTTTCGGTGATGACGGTGAAAACCTCTGACACATGCAGCTCCCGGAGACGGTCACAGCTTGTCTGTAAGCGGATGCCGGGAGCAGACAAGCCCGTCAGGGCGCGTCAGCGGGTGTTGGCGGGTGTCGGGGCTGGCTTAACTATGCGGCATCAGAGCAGATTGTACTGAGAGTGCACCATACCACCTTTTCAATTCATCATTTTTTTTTTATTCTTTTTTTTGATTTCGGTTTCCTTGAAATTTTTTTGATTCGGTAATCTCCGAACAGAAGGAAGAACGAAGGAAGGAGCACAGACTTAGATTGGTATATATACGCATATGTAGTGTTGAAGAAACATGAAATTGCCCAGTATTCTTAACCCAACTGCACAGAACAAAAACCTGCAGGAAACGAAGATAAATCATGTCGAAAGCTACATATAAGGAACGTGCTGCTACTCATCCTAGTCCTGTTGCTGCCAAGCTATTTAATATCATGCACGAAAAGCAAACAAACTTGTGTGCTTCATTGGATGTTCGTACCACCAAGGAATTACTGGAGTTAGTTGAAGCATTAGGTCCCAAAATTTGTTTACTAAAAACACATGTGGATATCTTGACTGATTTTTCCATGGAGGGCACAGTTAAGCCGCTAAAGGCATTATCCGCCAAGTACAATTTTTTACTCTTCGAAGACAGAAAATTTGCTGACATTGGTAATACAGTCAAATTGCAGTACTCTGCGGGTGTATACAGAATAGCAGAATGGGCAGACATTACGAATGCACACGGTGTGGTGGGCCCAGGTATTGTTAGCGGTTTGAAGCAGGCGGCAGAAGAAGTAACAAAGGAACCTAGAGGCCTTTTGATGTTAGCAGAATTGTCATGCAAGGGCTCCCTATCTACTGGAGAATATACTAAGGGTACTGTTGACATTGCGAAGAGCGACAAAGATTTTGTTATCGGCTTTATTGCTCAAAGAGACATGGGTGGAAGAGATGAAGGTTACGATTGGTTGATTATGACACCCGGTGTGGGTTTAGATGACAAGGGAGACGCATTGGGTCAACAGTATAGAACCGTGGATGATGTGGTCTCTACAGGATCTGACATTATTATTGTTGGAAGAGGACTATTTGCAAAGGGAAGGGATGCTAAGGTAGAGGGTGAACGTTACAGAAAAGCAGGCTGGGAAGCATATTTGAGAAGATGCGGCCAGCAAAACTAAAAAACTGTATTATAAGTAAATGCATGTATACTAAACTCACAAATTAGAGCTTCAATTTAATTATATCAGTTATTACCCTATGCGGTGTGAAATACCGCACAGATGCGTAAGGAGAAAATACCGCATCAGGAAATTGTAAGCGTTAATATTTTGTTAAAATTCGCGTTAAATTTTTGTTAAATCAGCTCATTTTTTAACCAATAGGCCGAAATCGGCAAAATCCCTTATAAATCAAAAGAATAGACCGAGATAGGGTTGAGTGTTGTTCCAGTTTGGAACAAGAGTCCACTATTAAAGAACGTGGACTCCAACGTCAAAGGGCGAAAAACCGTCTATCAGGGCGATGGCCCACTACGTGAACCATCACCCTAATCAAGTTTTTTGGGGTCGAGGTGCCGTAAAGCACTAAATCGGAACCCTAAAGGGAGCCCCCGATTTAGAGCTTGACGGGGAAAGCCGGCGAACGTGGCGAGAAAGGAAGGGAAGAAAGCGAAAGGAGCGGGCGCTAGGGCGCTGGCAAGTGTAGCGGTCACGCTGCGCGTAACCACCACACCCGCCGCGCTTAATGCGCCGCTACAGGGCGCGTCCATTCGCCATTCAGGCTGCGCAACTGTTGGGAAGGGCGATCGGTGCGGGCCTCTTCGCTATTACGCCAGCTGGCGAAAGGGGGATGTGCTGCAAGGCGATTAAGTTGGGTAACGCCAGGGTTTTCCCAGTCACGACGTTGTAAAACGACGGCCAGTGAGCGCGCGTAATACGACTCACTATAGGGCGAATTGGGTACCGGGCCCCCCCTCGAGGTCGACGGTATCGATAAGCTTGATATCGAATTCAGTCTACTAGTCAGTTCGAGTTTATCATTATCAATACTGCCATTTCAAAGAATACGTAAATAATTAATAGTAGTGATTTTCCTAACTTTATTTAGTCAAAAAATTAGCCTTTTAATTCTGCTGTAACCCGTACATGCCCAAAATAGGGGGCGGGTTACACAGAATATATAACATCGTAGGTGTCTGGGTGAACAGTTTATTCCTGGCATCCACTAAATATAATGGAGCCCGCTTTTTAAGCTGGCATCCAGAAAAAAAAAGAATCCCAGCACCAAAATATTGTTTTCTTCACCAACCATCAGTTCATAGGTCCATTCTCTTAGCGCAACTACAGAGAACAGGGGCACAAACAGGCAAAAAACGGGCACAACCTCAATGGAGTGATGCAACCTGCCTGGAGTAAATGATGACACAAGGCAATTGACCCACGCATGTATCTATCTCATTTTCTTACACCTTCTATTACCTTCTGCTCTCTCTGATTTGGAAAAAGCTGAAAAAAAAGGTTGAAACCAGTTCCCTGAAATTATTCCCCTACTTGACTAATAAGTATATAAAGACGGTAGGTATTGATTGTAATTCTGTAAATCTATTTCTTAAACTTCTTAAATTCTACTTTTATAGTTAGTCTTTTTTTTAGTTTTAAAACACCAAGAACTTAGTTTCGAATAAACACACATAAACAAACAAAAGATCTGATATGGATCGAATTAGATCTCGCCACC

**Plasmid pRB322**

**
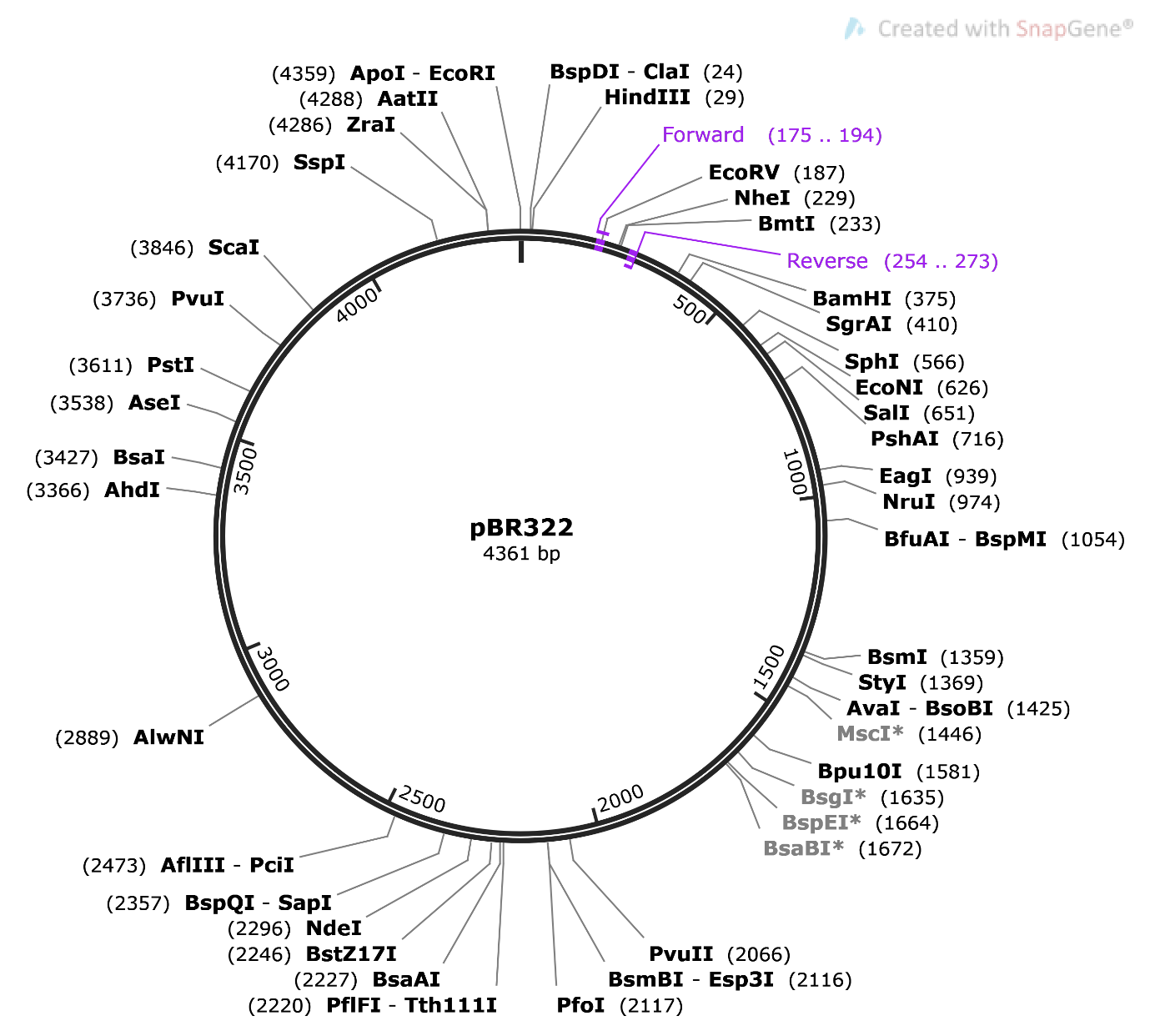
**

Plasmid sequence:

TTCTCATGTTTGACAGCTTATCATCGATAAGCTTTAATGCGGTAGTTTATCACAGTTAAATTGCTAACGCAGTCAGGCACCGTGTATGAAATCTAACAATGCGCTCATCGTCATCCTCGGCACCGTCACCCTGGATGCTGTAGGCATAGGCTTGGTTATGCCGGTACTGCCGGGCCTCTTGCGGGATATCGTCCATTCCGACAGCATCGCCAGTCACTATGGCGTGCTGCTAGCGCTATATGCGTTGATGCAATTTCTATGCGCACCCGTTCTCGGAGCACTGTCCGACCGCTTTGGCCGCCGCCCAGTCCTGCTCGCTTCGCTACTTGGAGCCACTATCGACTACGCGATCATGGCGACCACACCCGTCCTGTGGATCCTCTACGCCGGACGCATCGTGGCCGGCATCACCGGCGCCACAGGTGCGGTTGCTGGCGCCTATATCGCCGACATCACCGATGGGGAAGATCGGGCTCGCCACTTCGGGCTCATGAGCGCTTGTTTCGGCGTGGGTATGGTGGCAGGCCCCGTGGCCGGGGGACTGTTGGGCGCCATCTCCTTGCATGCACCATTCCTTGCGGCGGCGGTGCTCAACGGCCTCAACCTACTACTGGGCTGCTTCCTAATGCAGGAGTCGCATAAGGGAGAGCGTCGACCGATGCCCTTGAGAGCCTTCAACCCAGTCAGCTCCTTCCGGTGGGCGCGGGGCATGACTATCGTCGCCGCACTTATGACTGTCTTCTTTATCATGCAACTCGTAGGACAGGTGCCGGCAGCGCTCTGGGTCATTTTCGGCGAGGACCGCTTTCGCTGGAGCGCGACGATGATCGGCCTGTCGCTTGCGGTATTCGGAATCTTGCACGCCCTCGCTCAAGCCTTCGTCACTGGTCCCGCCACCAAACGTTTCGGCGAGAAGCAGGCCATTATCGCCGGCATGGCGGCCGACGCGCTGGGCTACGTCTTGCTGGCGTTCGCGACGCGAGGCTGGATGGCCTTCCCCATTATGATTCTTCTCGCTTCCGGCGGCATCGGGATGCCCGCGTTGCAGGCCATGCTGTCCAGGCAGGTAGATGACGACCATCAGGGACAGCTTCAAGGATCGCTCGCGGCTCTTACCAGCCTAACTTCGATCACTGGACCGCTGATCGTCACGGCGATTTATGCCGCCTCGGCGAGCACATGGAACGGGTTGGCATGGATTGTAGGCGCCGCCCTATACCTTGTCTGCCTCCCCGCGTTGCGTCGCGGTGCATGGAGCCGGGCCACCTCGACCTGAATGGAAGCCGGCGGCACCTCGCTAACGGATTCACCACTCCAAGAATTGGAGCCAATCAATTCTTGCGGAGAACTGTGAATGCGCAAACCAACCCTTGGCAGAACATATCCATCGCGTCCGCCATCTCCAGCAGCCGCACGCGGCGCATCTCGGGCAGCGTTGGGTCCTGGCCACGGGTGCGCATGATCGTGCTCCTGTCGTTGAGGACCCGGCTAGGCTGGCGGGGTTGCCTTACTGGTTAGCAGAATGAATCACCGATACGCGAGCGAACGTGAAGCGACTGCTGCTGCAAAACGTCTGCGACCTGAGCAACAACATGAATGGTCTTCGGTTTCCGTGTTTCGTAAAGTCTGGAAACGCGGAAGTCAGCGCCCTGCACCATTATGTTCCGGATCTGCATCGCAGGATGCTGCTGGCTACCCTGTGGAACACCTACATCTGTATTAACGAAGCGCTGGCATTGACCCTGAGTGATTTTTCTCTGGTCCCGCCGCATCCATACCGCCAGTTGTTTACCCTCACAACGTTCCAGTAACCGGGCATGTTCATCATCAGTAACCCGTATCGTGAGCATCCTCTCTCGTTTCATCGGTATCATTACCCCCATGAACAGAAATCCCCCTTACACGGAGGCATCAGTGACCAAACAGGAAAAAACCGCCCTTAACATGGCCCGCTTTATCAGAAGCCAGACATTAACGCTTCTGGAGAAACTCAACGAGCTGGACGCGGATGAACAGGCAGACATCTGTGAATCGCTTCACGACCACGCTGATGAGCTTTACCGCAGCTGCCTCGCGCGTTTCGGTGATGACGGTGAAAACCTCTGACACATGCAGCTCCCGGAGACGGTCACAGCTTGTCTGTAAGCGGATGCCGGGAGCAGACAAGCCCGTCAGGGCGCGTCAGCGGGTGTTGGCGGGTGTCGGGGCGCAGCCATGACCCAGTCACGTAGCGATAGCGGAGTGTATACTGGCTTAACTATGCGGCATCAGAGCAGATTGTACTGAGAGTGCACCATATGCGGTGTGAAATACCGCACAGATGCGTAAGGAGAAAATACCGCATCAGGCGCTCTTCCGCTTCCTCGCTCACTGACTCGCTGCGCTCGGTCGTTCGGCTGCGGCGAGCGGTATCAGCTCACTCAAAGGCGGTAATACGGTTATCCACAGAATCAGGGGATAACGCAGGAAAGAACATGTGAGCAAAAGGCCAGCAAAAGGCCAGGAACCGTAAAAAGGCCGCGTTGCTGGCGTTTTTCCATAGGCTCCGCCCCCCTGACGAGCATCACAAAAATCGACGCTCAAGTCAGAGGTGGCGAAACCCGACAGGACTATAAAGATACCAGGCGTTTCCCCCTGGAAGCTCCCTCGTGCGCTCTCCTGTTCCGACCCTGCCGCTTACCGGATACCTGTCCGCCTTTCTCCCTTCGGGAAGCGTGGCGCTTTCTCATAGCTCACGCTGTAGGTATCTCAGTTCGGTGTAGGTCGTTCGCTCCAAGCTGGGCTGTGTGCACGAACCCCCCGTTCAGCCCGACCGCTGCGCCTTATCCGGTAACTATCGTCTTGAGTCCAACCCGGTAAGACACGACTTATCGCCACTGGCAGCAGCCACTGGTAACAGGATTAGCAGAGCGAGGTATGTAGGCGGTGCTACAGAGTTCTTGAAGTGGTGGCCTAACTACGGCTACACTAGAAGGACAGTATTTGGTATCTGCGCTCTGCTGAAGCCAGTTACCTTCGGAAAAAGAGTTGGTAGCTCTTGATCCGGCAAACAAACCACCGCTGGTAGCGGTGGTTTTTTTGTTTGCAAGCAGCAGATTACGCGCAGAAAAAAAGGATCTCAAGAAGATCCTTTGATCTTTTCTACGGGGTCTGACGCTCAGTGGAACGAAAACTCACGTTAAGGGATTTTGGTCATGAGATTATCAAAAAGGATCTTCACCTAGATCCTTTTAAATTAAAAATGAAGTTTTAAATCAATCTAAAGTATATATGAGTAAACTTGGTCTGACAGTTACCAATGCTTAATCAGTGAGGCACCTATCTCAGCGATCTGTCTATTTCGTTCATCCATAGTTGCCTGACTCCCCGTCGTGTAGATAACTACGATACGGGAGGGCTTACCATCTGGCCCCAGTGCTGCAATGATACCGCGAGACCCACGCTCACCGGCTCCAGATTTATCAGCAATAAACCAGCCAGCCGGAAGGGCCGAGCGCAGAAGTGGTCCTGCAACTTTATCCGCCTCCATCCAGTCTATTAATTGTTGCCGGGAAGCTAGAGTAAGTAGTTCGCCAGTTAATAGTTTGCGCAACGTTGTTGCCATTGCTGCAGGCATCGTGGTGTCACGCTCGTCGTTTGGTATGGCTTCATTCAGCTCCGGTTCCCAACGATCAAGGCGAGTTACATGATCCCCCATGTTGTGCAAAAAAGCGGTTAGCTCCTTCGGTCCTCCGATCGTTGTCAGAAGTAAGTTGGCCGCAGTGTTATCACTCATGGTTATGGCAGCACTGCATAATTCTCTTACTGTCATGCCATCCGTAAGATGCTTTTCTGTGACTGGTGAGTACTCAACCAAGTCATTCTGAGAATAGTGTATGCGGCGACCGAGTTGCTCTTGCCCGGCGTCAACACGGGATAATACCGCGCCACATAGCAGAACTTTAAAAGTGCTCATCATTGGAAAACGTTCTTCGGGGCGAAAACTCTCAAGGATCTTACCGCTGTTGAGATCCAGTTCGATGTAACCCACTCGTGCACCCAACTGATCTTCAGCATCTTTTACTTTCACCAGCGTTTCTGGGTGAGCAAAAACAGGAAGGCAAAATGCCGCAAAAAAGGGAATAAGGGCGACACGGAAATGTTGAATACTCATACTCTTCCTTTTTCAATATTATTGAAGCATTTATCAGGGTTATTGTCTCATGAGCGGATACATATTTGAATGTATTTAGAAAAATAAACAAATAGGGGTTCCGCGCACATTTCCCCGAAAAGTGCCACCTGACGTCTAAGAAACCATTATTATCATGACATTAACCTATAAAAATAGGCGTATCACGAGGCCCTTTCGTCTTCAAGAA

**Supplementary Figure 2**

**KRAS standard curves at 60, 62 and 63 °C.**

**
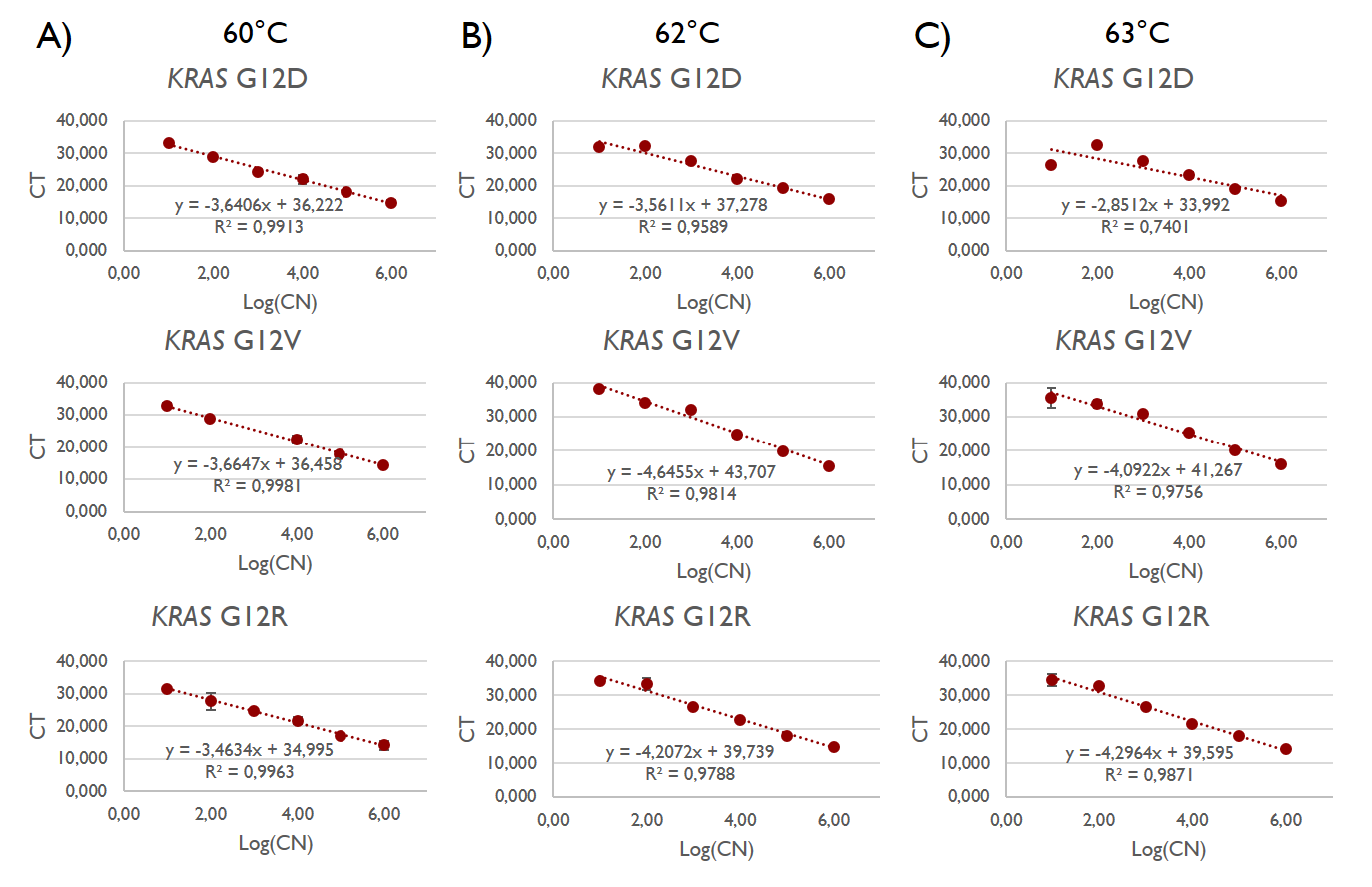
**

**Supplementary figure 2, optimization of primer annealing temperature**
**A-C**) Depicts the qPCR standard threshold cycle (CT) curves for the detection of the three tested KRAS SNP types (G12D, G12V, and G12R) at different annealing temperatures: **A**) regular 60 °C, **B**) increased 62 °C and **C)** 63 °C. Log(CN) = logarithm of the copy number of plasmids used for the standard curve.

**Supplementary Figure 3**

**A) Primer mix: F: 150 nM *KRAS*^wt^, R: 150 nM *KRAS*^G12D^ reverse, R: 300 nM *KRAS*^wt^ dd-primer**

| **Plasmid (*KRAS*^wt^)** | **Mean Ct(*KRAS*^wt^)** | **Plasmid (*KRAS*^G12D^)** | **Mean**  **Ct(*KRAS*^G12D^)** | **Fold change** |
| --- | --- | --- | --- | --- |
| 10^6^ copies | 24.75 | 10^6^ copies | 16.77 | 0.004 |
| 10^5^ copies | 28.65 | 10^5^ copies | 20.52 | 0.004 |
| 10^4^ copies | 33.63 | 10^4^ copies | 24.22 | 0.001 |
| 10^3^ copies | 33.03 | 10^3^ copies | 27.54 | 0.022 |
| 10^2^ copies | 31.91 | 10^2^ copies | 31.87 | 1.548 |
| 10 copies | 34.35 | 10 copies | 33.50 | 0.555 |

**B) Primer mix: F: 100 nM *KRAS*^wt^, R: 100 nM *KRAS*^G12D^ reverse, R: 400 nM *KRAS*^wt^ dd-primer**

| **Plasmid (*KRAS*^wt^)** | **Mean Ct(*KRAS*^wt^)** | **Plasmid (*KRAS*^G12D^)** | **Mean**  **Ct(*KRAS*^G12D^)** | **Fold change** |
| --- | --- | --- | --- | --- |
| 10^6^ copies | 25.75 | 10^6^ copies | 16.23 | 0.001 |
| 10^5^ copies | 28.97 | 10^5^ copies | 19.72 | 0.002 |
| 10^4^ copies | 32.42 | 10^4^ copies | 24.40 | 0.006 |
| 10^3^ copies | 33.92 | 10^3^ copies | 28.92 | 0.020 |
| 10^2^ copies | below detection | 10^2^ copies | 32.72 | - |
| 10 copies | 33.42 | 10 copies | below detection | - |

**C)**

**Supplementary figure 3. WT allele blocking does not improve mutant allele qPCR detection**qPCR was run for *KRAS*^wt^-plasmids and *KRAS*^G12D^-plasmids (**Supplementary figure 4**). The same mixed primerset was used for all measurements though at different concentration ratios: Forward (F) primer: *KRAS*^wt^, reverse (R) primer 1: *KRAS*^G12D^, reverse primer 2: KRAS^wt^ dd-primer (3´-end. ddC).
Fold change was determined as 2^-ΔΔCt = 2^ -(Ct(*KRAS*^wt^)- Ct(*KRAS*^G12D^)), as each experimental Ct was determined on the same qPCR run for figure A and B respectively. Each sample was tested in triplicate and Ct-values above 35 were designated as being below the detection limit (not detected).
**A**) A table of measured Ct-values for wt KRAS and G12D mutant allele detection in the presence of dd-primer at a relative reverse primer ratio of 2x. **B**) A table of measured Ct-values for wt KRAS and G12D mutant allele detection in the presence of dd-primer at a relative reverse primer ratio of 4x.

**C**) Barplot of the fold change results from table A (left side) and B (right side).
Increasing the relative dd-primer concentration did not lead to any marked improvement in fold change mutant specific detection rates.

**Supplementary Figure 4**

**pUC57 KRAS wt plasmid**


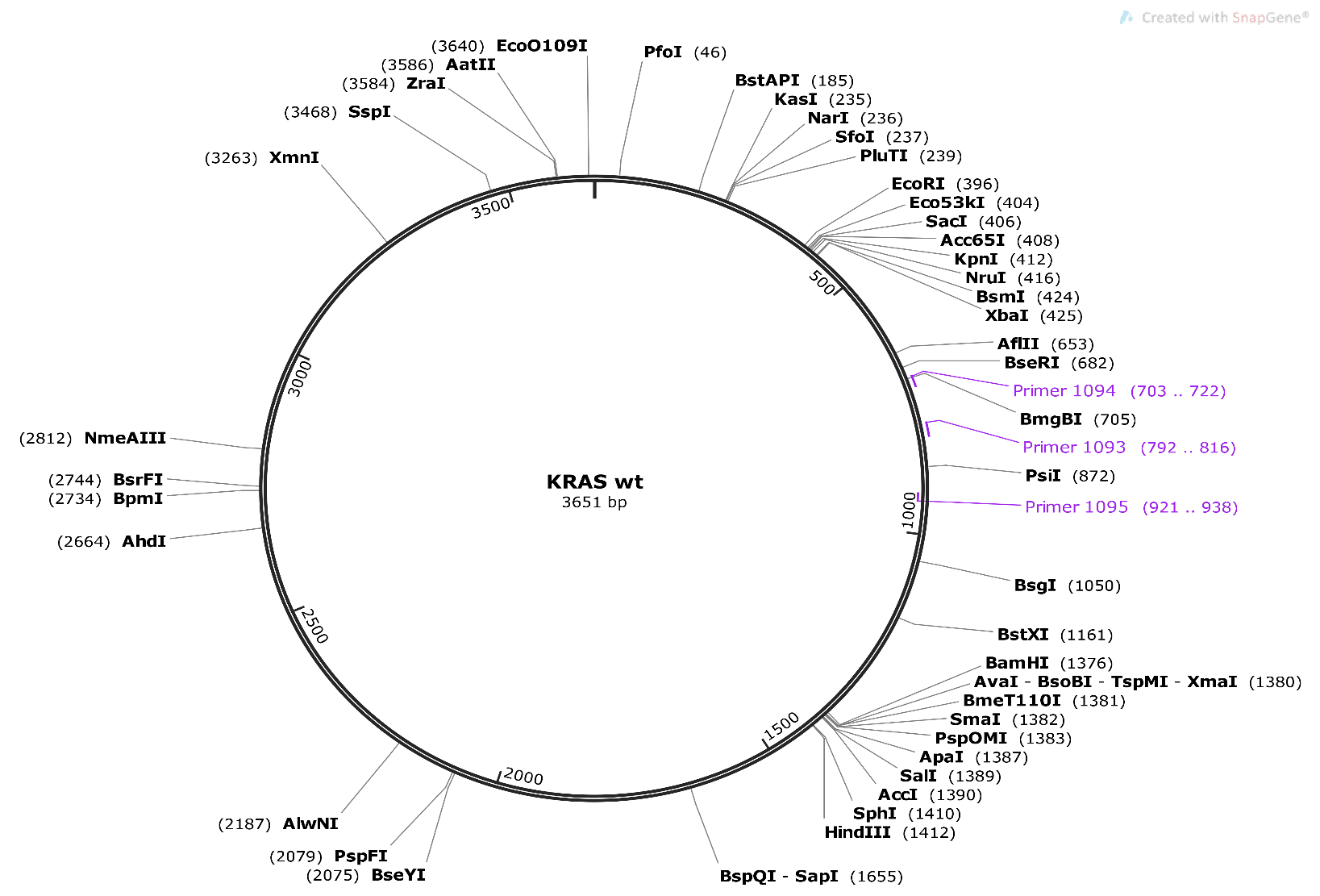

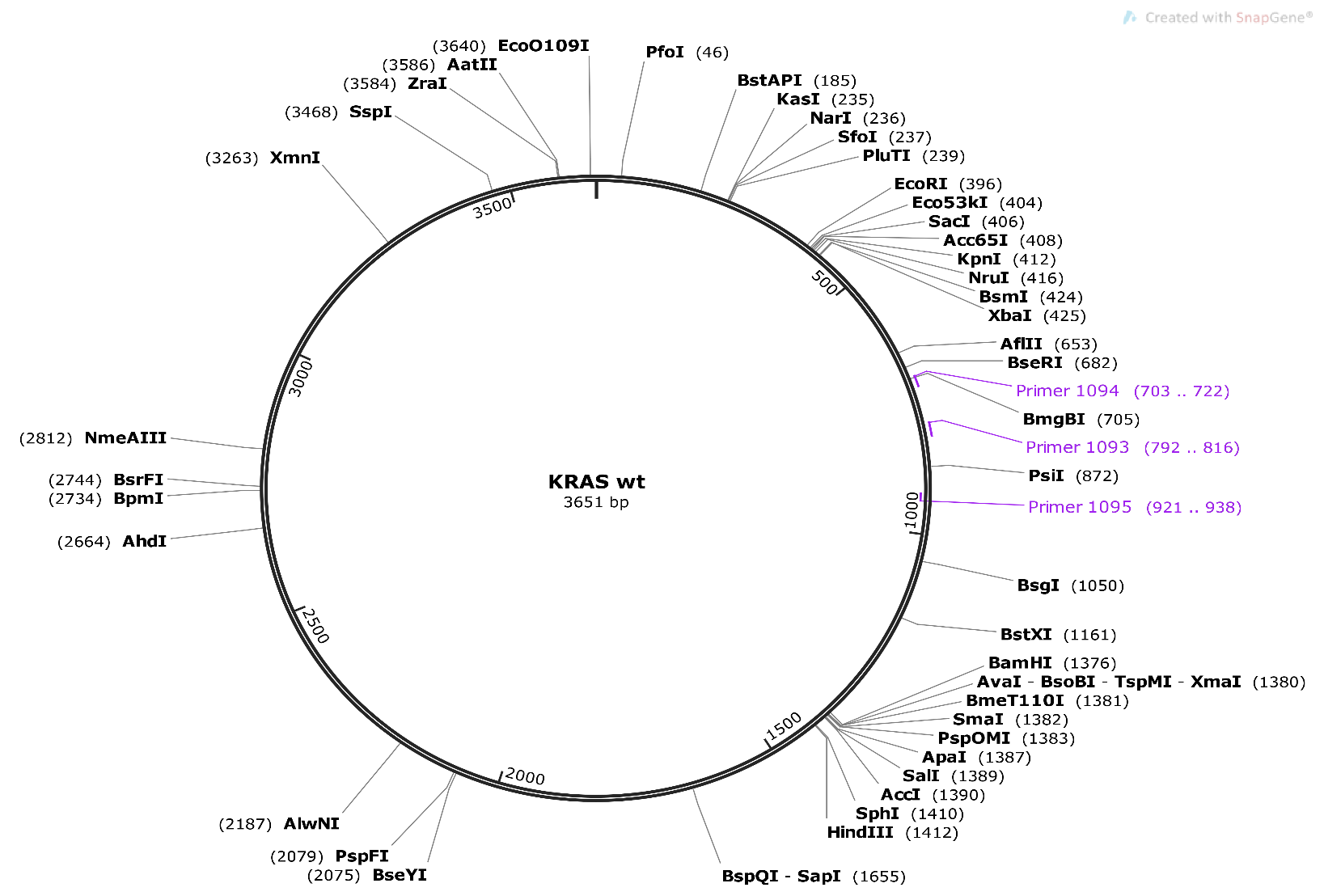

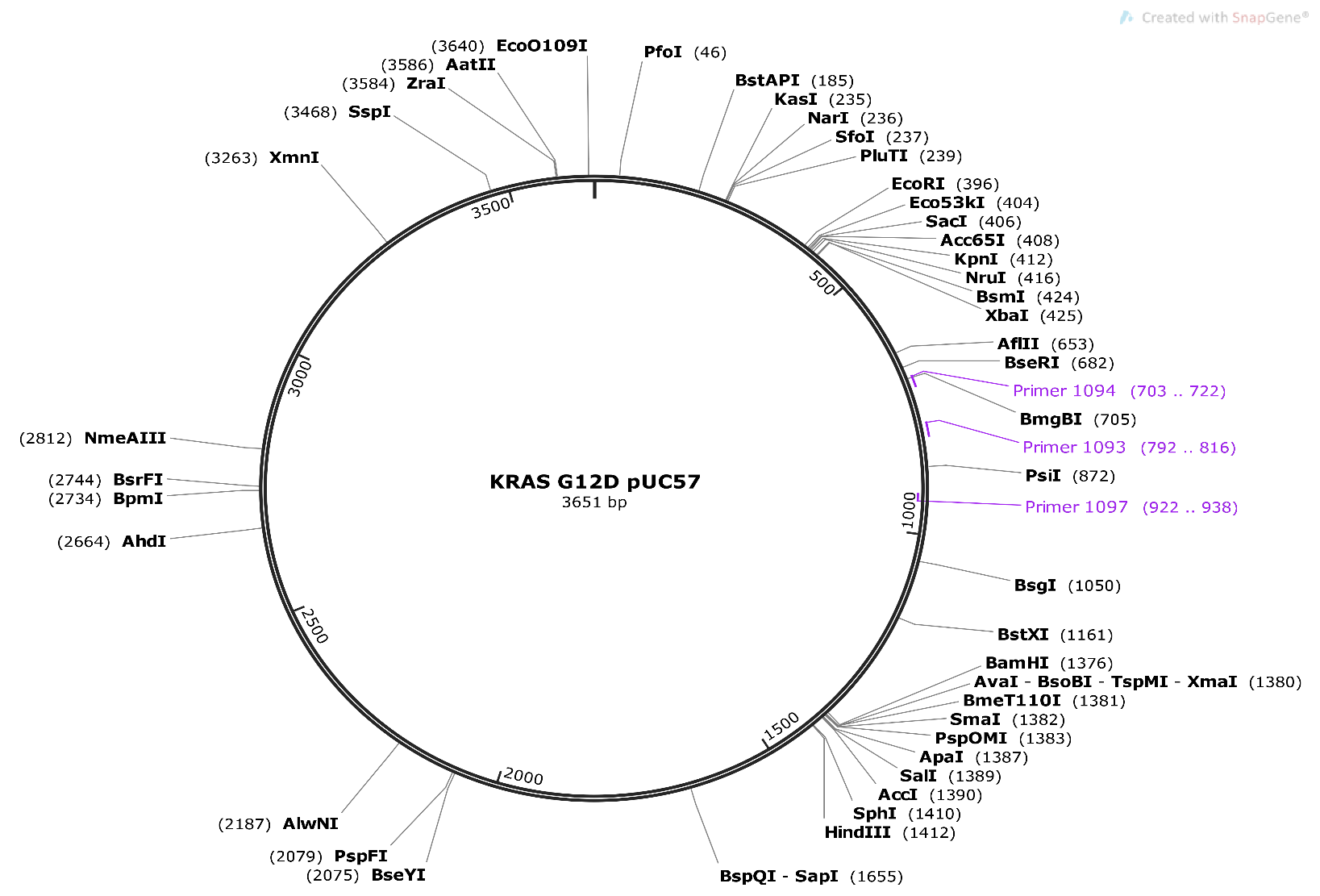


Plasmid sequence:

TCGCGCGTTTCGGTGATGACGGTGAAAACCTCTGACACATGCAGCTCCCGGAGACGGTCACAGCTTGTCTGTAAGCGGATGCCGGGAGCAGACAAGCCCGTCAGGGCGCGTCAGCGGGTGTTGGCGGGTGTCGGGGCTGGCTTAACTATGCGGCATCAGAGCAGATTGTACTGAGAGTGCACCATATGCGGTGTGAAATACCGCACAGATGCGTAAGGAGAAAATACCGCATCAGGCGCCATTCGCCATTCAGGCTGCGCAACTGTTGGGAAGGGCGATCGGTGCGGGCCTCTTCGCTATTACGCCAGCTGGCGAAAGGGGGATGTGCTGCAAGGCGATTAAGTTGGGTAACGCCAGGGTTTTCCCAGTCACGACGTTGTAAAACGACGGCCAGTGAATTCGAGCTCGGTACCTCGCGAATGCATCTAGATTCCACTAGGAAAACTGTAACAATAAGAGTGGAGATAGCTGTCAGCAACTTTTGTGAGGGTGTGCTACAGGGTGTAGAGCACTGTGAAGTCTCTACATGAGTGAAGTCATGATATGATCCTTTGAGAGCCTTTAGCCGCCGCAGAACAGCAGTCTGGCTATTTAGATAGAACAACTTGATTTTAAGATAAAAGAACTGTCTATGTAGCATTTATGCATTTTTCTTAAGCGTCGATGGAGGAGTTTGTAAATGAAGTACAGTTCATTACGATACACGTCTGCAGTCAACTGGAATTTTCATGATTGAATTTTGTAAGGTATTTTGAAATAATTTTTCATATAAAGGTGAGTTTGTATTAAAAGGTACTGGTGGAGTATTTGATAGTGTATTAACCTTATGTGTGACATGTTCTAATATAGTCACATTTTCATTATTTTTATTATAAGGCCTGCTGAAAATGACTGAATATAAACTTGTGGTAGTTGGAGCTGGTGGCGTAGGCAAGAGTGCCTTGACGATACAGCTAATTCAGAATCATTTTGTGGACGAATATGATCCAACAATAGAGGTAAATCTTGTTTTAATATGCATATTACTGGTGCAGGACCATTCTTTGATACAGATAAAGGTTTCTCTGACCATTTTCATGAGTACTTATTACAAGATAATTATGCTGAAAGTTAAGTTATCTGAAATGTACCTTGGGTTTCAAGTTATATGTAACCATTAATATGGGAACTTTACTTTCCTTGGGAGTATGTCAGGGTCCATGATGTTCACTCTCTGTGCATTTTGATTGGAAGTGTATTTCAGAGTTTCGTGAGAGGGTAGAAATTTGTATCCTATCTGGACCTAAAAGACAATCTTTTTATTGTAACTTTTATTTTTATGGGTTTCTTGGTATTGTGACATCATATGTAAAGGTTAGATTTAATTGTACTAATCGGATCCCGGGCCCGTCGACTGCAGAGGCCTGCATGCAAGCTTGGCGTAATCATGGTCATAGCTGTTTCCTGTGTGAAATTGTTATCCGCTCACAATTCCACACAACATACGAGCCGGAAGCATAAAGTGTAAAGCCTGGGGTGCCTAATGAGTGAGCTAACTCACATTAATTGCGTTGCGCTCACTGCCCGCTTTCCAGTCGGGAAACCTGTCGTGCCAGCTGCATTAATGAATCGGCCAACGCGCGGGGAGAGGCGGTTTGCGTATTGGGCGCTCTTCCGCTTCCTCGCTCACTGACTCGCTGCGCTCGGTCGTTCGGCTGCGGCGAGCGGTATCAGCTCACTCAAAGGCGGTAATACGGTTATCCACAGAATCAGGGGATAACGCAGGAAAGAACATGTGAGCAAAAGGCCAGCAAAAGGCCAGGAACCGTAAAAAGGCCGCGTTGCTGGCGTTTTTCCATAGGCTCCGCCCCCCTGACGAGCATCACAAAAATCGACGCTCAAGTCAGAGGTGGCGAAACCCGACAGGACTATAAAGATACCAGGCGTTTCCCCCTGGAAGCTCCCTCGTGCGCTCTCCTGTTCCGACCCTGCCGCTTACCGGATACCTGTCCGCCTTTCTCCCTTCGGGAAGCGTGGCGCTTTCTCATAGCTCACGCTGTAGGTATCTCAGTTCGGTGTAGGTCGTTCGCTCCAAGCTGGGCTGTGTGCACGAACCCCCCGTTCAGCCCGACCGCTGCGCCTTATCCGGTAACTATCGTCTTGAGTCCAACCCGGTAAGACACGACTTATCGCCACTGGCAGCAGCCACTGGTAACAGGATTAGCAGAGCGAGGTATGTAGGCGGTGCTACAGAGTTCTTGAAGTGGTGGCCTAACTACGGCTACACTAGAAGAACAGTATTTGGTATCTGCGCTCTGCTGAAGCCAGTTACCTTCGGAAAAAGAGTTGGTAGCTCTTGATCCGGCAAACAAACCACCGCTGGTAGCGGTGGTTTTTTTGTTTGCAAGCAGCAGATTACGCGCAGAAAAAAAGGATCTCAAGAAGATCCTTTGATCTTTTCTACGGGGTCTGACGCTCAGTGGAACGAAAACTCACGTTAAGGGATTTTGGTCATGAGATTATCAAAAAGGATCTTCACCTAGATCCTTTTAAATTAAAAATGAAGTTTTAAATCAATCTAAAGTATATATGAGTAAACTTGGTCTGACAGTTACCAATGCTTAATCAGTGAGGCACCTATCTCAGCGATCTGTCTATTTCGTTCATCCATAGTTGCCTGACTCCCCGTCGTGTAGATAACTACGATACGGGAGGGCTTACCATCTGGCCCCAGTGCTGCAATGATACCGCGAGATCCACGCTCACCGGCTCCAGATTTATCAGCAATAAACCAGCCAGCCGGAAGGGCCGAGCGCAGAAGTGGTCCTGCAACTTTATCCGCCTCCATCCAGTCTATTAATTGTTGCCGGGAAGCTAGAGTAAGTAGTTCGCCAGTTAATAGTTTGCGCAACGTTGTTGCCATTGCTACAGGCATCGTGGTGTCACGCTCGTCGTTTGGTATGGCTTCATTCAGCTCCGGTTCCCAACGATCAAGGCGAGTTACATGATCCCCCATGTTGTGCAAAAAAGCGGTTAGCTCCTTCGGTCCTCCGATCGTTGTCAGAAGTAAGTTGGCCGCAGTGTTATCACTCATGGTTATGGCAGCACTGCATAATTCTCTTACTGTCATGCCATCCGTAAGATGCTTTTCTGTGACTGGTGAGTACTCAACCAAGTCATTCTGAGAATAGTGTATGCGGCGACCGAGTTGCTCTTGCCCGGCGTCAATACGGGATAATACCGCGCCACATAGCAGAACTTTAAAAGTGCTCATCATTGGAAAACGTTCTTCGGGGCGAAAACTCTCAAGGATCTTACCGCTGTTGAGATCCAGTTCGATGTAACCCACTCGTGCACCCAACTGATCTTCAGCATCTTTTACTTTCACCAGCGTTTCTGGGTGAGCAAAAACAGGAAGGCAAAATGCCGCAAAAAAGGGAATAAGGGCGACACGGAAATGTTGAATACTCATACTCTTCCTTTTTCAATATTATTGAAGCATTTATCAGGGTTATTGTCTCATGAGCGGATACATATTTGAATGTATTTAGAAAAATAAACAAATAGGGGTTCCGCGCACATTTCCCCGAAAAGTGCCACCTGACGTCTAAGAAACCATTATTATCATGACATTAACCTATAAAAATAGGCGTATCACGAGGCCCTTTCGTC

**pUC57 KRAS G12V plasmid**

**
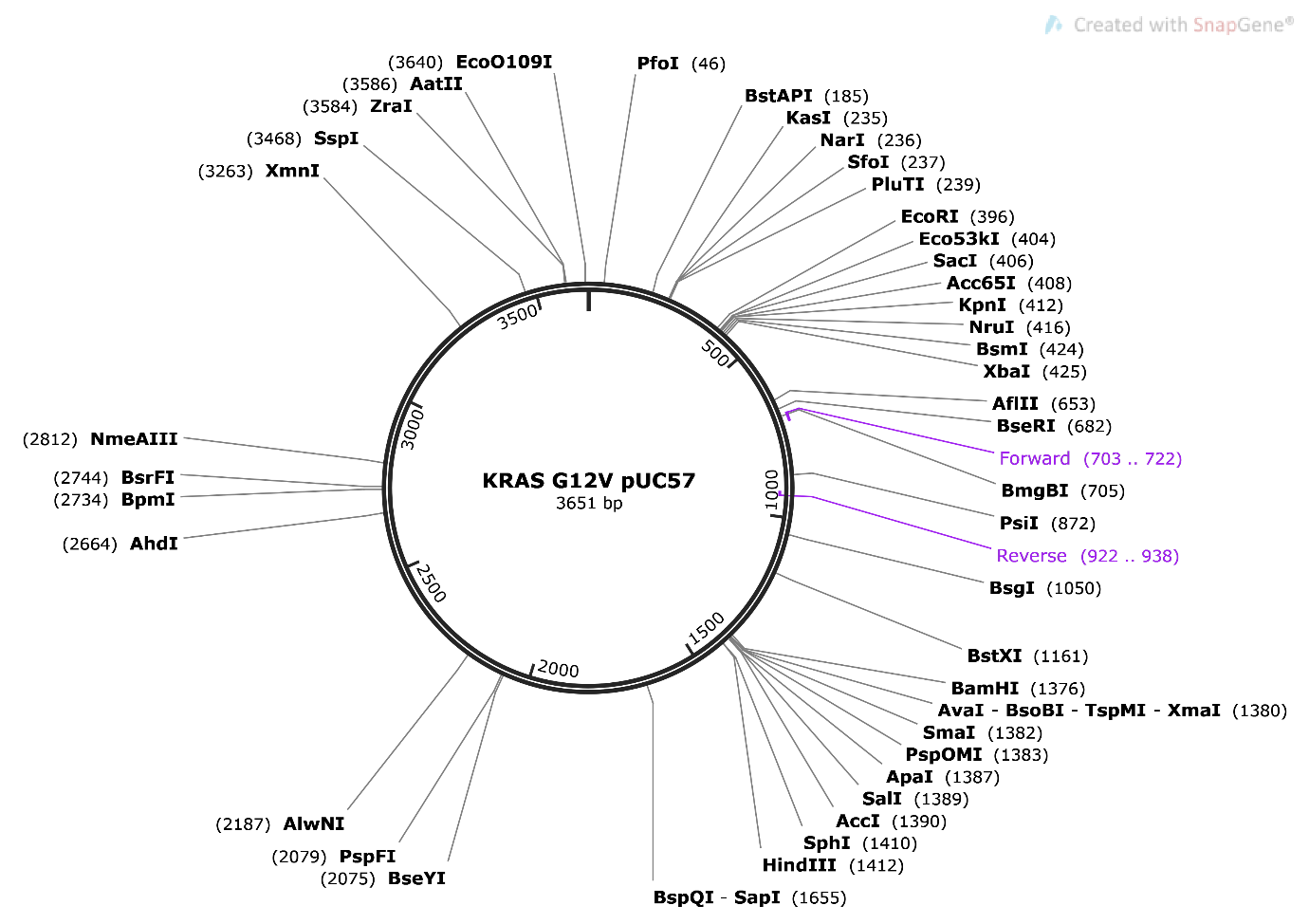
**

Plasmid sequence:

TCGCGCGTTTCGGTGATGACGGTGAAAACCTCTGACACATGCAGCTCCCGGAGACGGTCACAGCTTGTCTGTAAGCGGATGCCGGGAGCAGACAAGCCCGTCAGGGCGCGTCAGCGGGTGTTGGCGGGTGTCGGGGCTGGCTTAACTATGCGGCATCAGAGCAGATTGTACTGAGAGTGCACCATATGCGGTGTGAAATACCGCACAGATGCGTAAGGAGAAAATACCGCATCAGGCGCCATTCGCCATTCAGGCTGCGCAACTGTTGGGAAGGGCGATCGGTGCGGGCCTCTTCGCTATTACGCCAGCTGGCGAAAGGGGGATGTGCTGCAAGGCGATTAAGTTGGGTAACGCCAGGGTTTTCCCAGTCACGACGTTGTAAAACGACGGCCAGTGAATTCGAGCTCGGTACCTCGCGAATGCATCTAGATTCCACTAGGAAAACTGTAACAATAAGAGTGGAGATAGCTGTCAGCAACTTTTGTGAGGGTGTGCTACAGGGTGTAGAGCACTGTGAAGTCTCTACATGAGTGAAGTCATGATATGATCCTTTGAGAGCCTTTAGCCGCCGCAGAACAGCAGTCTGGCTATTTAGATAGAACAACTTGATTTTAAGATAAAAGAACTGTCTATGTAGCATTTATGCATTTTTCTTAAGCGTCGATGGAGGAGTTTGTAAATGAAGTACAGTTCATTACGATACACGTCTGCAGTCAACTGGAATTTTCATGATTGAATTTTGTAAGGTATTTTGAAATAATTTTTCATATAAAGGTGAGTTTGTATTAAAAGGTACTGGTGGAGTATTTGATAGTGTATTAACCTTATGTGTGACATGTTCTAATATAGTCACATTTTCATTATTTTTATTATAAGGCCTGCTGAAAATGACTGAATATAAACTTGTGGTAGTTGGAGCTGTTGGCGTAGGCAAGAGTGCCTTGACGATACAGCTAATTCAGAATCATTTTGTGGACGAATATGATCCAACAATAGAGGTAAATCTTGTTTTAATATGCATATTACTGGTGCAGGACCATTCTTTGATACAGATAAAGGTTTCTCTGACCATTTTCATGAGTACTTATTACAAGATAATTATGCTGAAAGTTAAGTTATCTGAAATGTACCTTGGGTTTCAAGTTATATGTAACCATTAATATGGGAACTTTACTTTCCTTGGGAGTATGTCAGGGTCCATGATGTTCACTCTCTGTGCATTTTGATTGGAAGTGTATTTCAGAGTTTCGTGAGAGGGTAGAAATTTGTATCCTATCTGGACCTAAAAGACAATCTTTTTATTGTAACTTTTATTTTTATGGGTTTCTTGGTATTGTGACATCATATGTAAAGGTTAGATTTAATTGTACTAATCGGATCCCGGGCCCGTCGACTGCAGAGGCCTGCATGCAAGCTTGGCGTAATCATGGTCATAGCTGTTTCCTGTGTGAAATTGTTATCCGCTCACAATTCCACACAACATACGAGCCGGAAGCATAAAGTGTAAAGCCTGGGGTGCCTAATGAGTGAGCTAACTCACATTAATTGCGTTGCGCTCACTGCCCGCTTTCCAGTCGGGAAACCTGTCGTGCCAGCTGCATTAATGAATCGGCCAACGCGCGGGGAGAGGCGGTTTGCGTATTGGGCGCTCTTCCGCTTCCTCGCTCACTGACTCGCTGCGCTCGGTCGTTCGGCTGCGGCGAGCGGTATCAGCTCACTCAAAGGCGGTAATACGGTTATCCACAGAATCAGGGGATAACGCAGGAAAGAACATGTGAGCAAAAGGCCAGCAAAAGGCCAGGAACCGTAAAAAGGCCGCGTTGCTGGCGTTTTTCCATAGGCTCCGCCCCCCTGACGAGCATCACAAAAATCGACGCTCAAGTCAGAGGTGGCGAAACCCGACAGGACTATAAAGATACCAGGCGTTTCCCCCTGGAAGCTCCCTCGTGCGCTCTCCTGTTCCGACCCTGCCGCTTACCGGATACCTGTCCGCCTTTCTCCCTTCGGGAAGCGTGGCGCTTTCTCATAGCTCACGCTGTAGGTATCTCAGTTCGGTGTAGGTCGTTCGCTCCAAGCTGGGCTGTGTGCACGAACCCCCCGTTCAGCCCGACCGCTGCGCCTTATCCGGTAACTATCGTCTTGAGTCCAACCCGGTAAGACACGACTTATCGCCACTGGCAGCAGCCACTGGTAACAGGATTAGCAGAGCGAGGTATGTAGGCGGTGCTACAGAGTTCTTGAAGTGGTGGCCTAACTACGGCTACACTAGAAGAACAGTATTTGGTATCTGCGCTCTGCTGAAGCCAGTTACCTTCGGAAAAAGAGTTGGTAGCTCTTGATCCGGCAAACAAACCACCGCTGGTAGCGGTGGTTTTTTTGTTTGCAAGCAGCAGATTACGCGCAGAAAAAAAGGATCTCAAGAAGATCCTTTGATCTTTTCTACGGGGTCTGACGCTCAGTGGAACGAAAACTCACGTTAAGGGATTTTGGTCATGAGATTATCAAAAAGGATCTTCACCTAGATCCTTTTAAATTAAAAATGAAGTTTTAAATCAATCTAAAGTATATATGAGTAAACTTGGTCTGACAGTTACCAATGCTTAATCAGTGAGGCACCTATCTCAGCGATCTGTCTATTTCGTTCATCCATAGTTGCCTGACTCCCCGTCGTGTAGATAACTACGATACGGGAGGGCTTACCATCTGGCCCCAGTGCTGCAATGATACCGCGAGATCCACGCTCACCGGCTCCAGATTTATCAGCAATAAACCAGCCAGCCGGAAGGGCCGAGCGCAGAAGTGGTCCTGCAACTTTATCCGCCTCCATCCAGTCTATTAATTGTTGCCGGGAAGCTAGAGTAAGTAGTTCGCCAGTTAATAGTTTGCGCAACGTTGTTGCCATTGCTACAGGCATCGTGGTGTCACGCTCGTCGTTTGGTATGGCTTCATTCAGCTCCGGTTCCCAACGATCAAGGCGAGTTACATGATCCCCCATGTTGTGCAAAAAAGCGGTTAGCTCCTTCGGTCCTCCGATCGTTGTCAGAAGTAAGTTGGCCGCAGTGTTATCACTCATGGTTATGGCAGCACTGCATAATTCTCTTACTGTCATGCCATCCGTAAGATGCTTTTCTGTGACTGGTGAGTACTCAACCAAGTCATTCTGAGAATAGTGTATGCGGCGACCGAGTTGCTCTTGCCCGGCGTCAATACGGGATAATACCGCGCCACATAGCAGAACTTTAAAAGTGCTCATCATTGGAAAACGTTCTTCGGGGCGAAAACTCTCAAGGATCTTACCGCTGTTGAGATCCAGTTCGATGTAACCCACTCGTGCACCCAACTGATCTTCAGCATCTTTTACTTTCACCAGCGTTTCTGGGTGAGCAAAAACAGGAAGGCAAAATGCCGCAAAAAAGGGAATAAGGGCGACACGGAAATGTTGAATACTCATACTCTTCCTTTTTCAATATTATTGAAGCATTTATCAGGGTTATTGTCTCATGAGCGGATACATATTTGAATGTATTTAGAAAAATAAACAAATAGGGGTTCCGCGCACATTTCCCCGAAAAGTGCCACCTGACGTCTAAGAAACCATTATTATCATGACATTAACCTATAAAAATAGGCGTATCACGAGGCCCTTTCGTC

**pUC57 KRAS G12R plasmid**


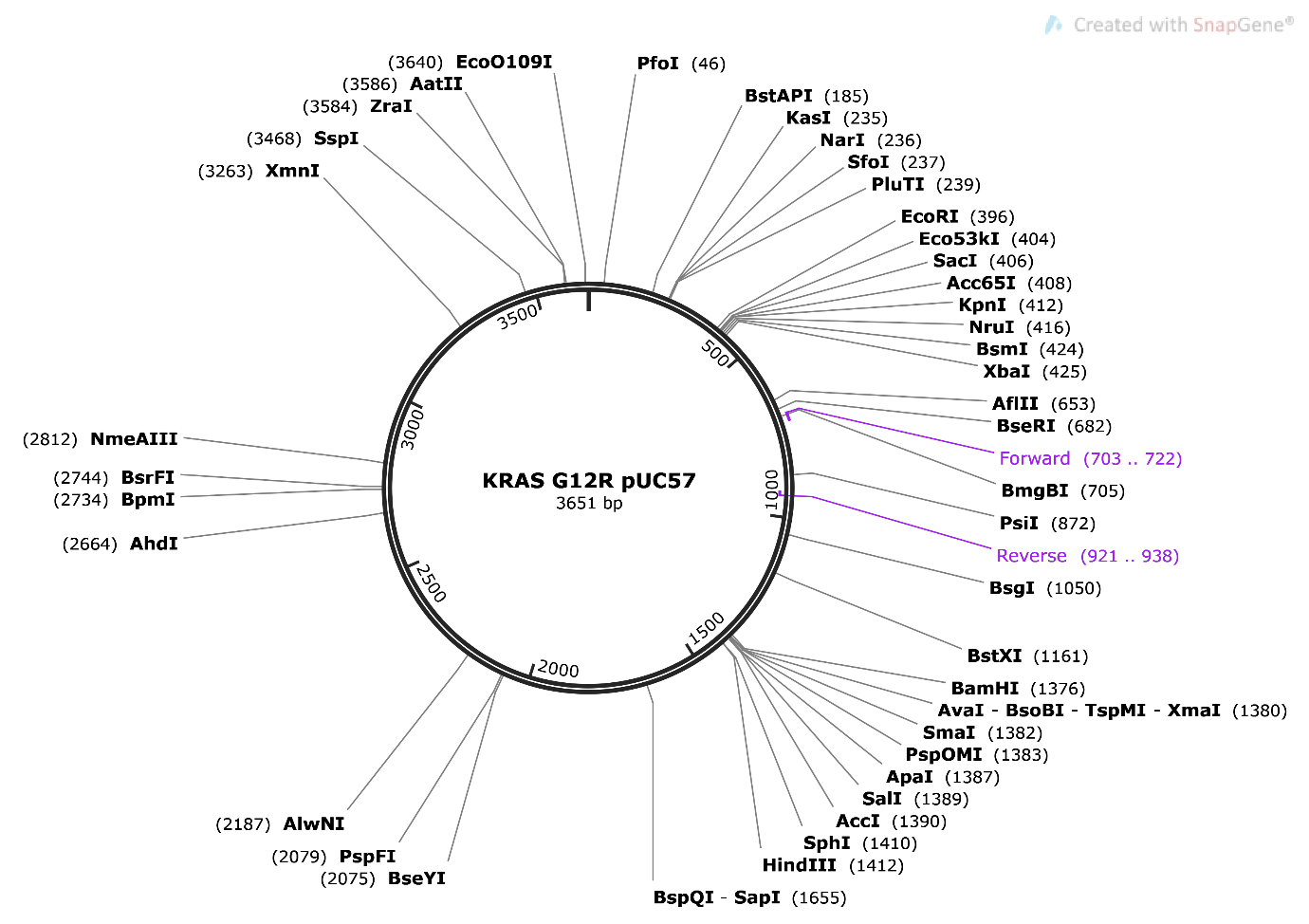


Plasmid sequence:

TCGCGCGTTTCGGTGATGACGGTGAAAACCTCTGACACATGCAGCTCCCGGAGACGGTCACAGCTTGTCTGTAAGCGGATGCCGGGAGCAGACAAGCCCGTCAGGGCGCGTCAGCGGGTGTTGGCGGGTGTCGGGGCTGGCTTAACTATGCGGCATCAGAGCAGATTGTACTGAGAGTGCACCATATGCGGTGTGAAATACCGCACAGATGCGTAAGGAGAAAATACCGCATCAGGCGCCATTCGCCATTCAGGCTGCGCAACTGTTGGGAAGGGCGATCGGTGCGGGCCTCTTCGCTATTACGCCAGCTGGCGAAAGGGGGATGTGCTGCAAGGCGATTAAGTTGGGTAACGCCAGGGTTTTCCCAGTCACGACGTTGTAAAACGACGGCCAGTGAATTCGAGCTCGGTACCTCGCGAATGCATCTAGATTCCACTAGGAAAACTGTAACAATAAGAGTGGAGATAGCTGTCAGCAACTTTTGTGAGGGTGTGCTACAGGGTGTAGAGCACTGTGAAGTCTCTACATGAGTGAAGTCATGATATGATCCTTTGAGAGCCTTTAGCCGCCGCAGAACAGCAGTCTGGCTATTTAGATAGAACAACTTGATTTTAAGATAAAAGAACTGTCTATGTAGCATTTATGCATTTTTCTTAAGCGTCGATGGAGGAGTTTGTAAATGAAGTACAGTTCATTACGATACACGTCTGCAGTCAACTGGAATTTTCATGATTGAATTTTGTAAGGTATTTTGAAATAATTTTTCATATAAAGGTGAGTTTGTATTAAAAGGTACTGGTGGAGTATTTGATAGTGTATTAACCTTATGTGTGACATGTTCTAATATAGTCACATTTTCATTATTTTTATTATAAGGCCTGCTGAAAATGACTGAATATAAACTTGTGGTAGTTGGAGCTAGAGGCGTAGGCAAGAGTGCCTTGACGATACAGCTAATTCAGAATCATTTTGTGGACGAATATGATCCAACAATAGAGGTAAATCTTGTTTTAATATGCATATTACTGGTGCAGGACCATTCTTTGATACAGATAAAGGTTTCTCTGACCATTTTCATGAGTACTTATTACAAGATAATTATGCTGAAAGTTAAGTTATCTGAAATGTACCTTGGGTTTCAAGTTATATGTAACCATTAATATGGGAACTTTACTTTCCTTGGGAGTATGTCAGGGTCCATGATGTTCACTCTCTGTGCATTTTGATTGGAAGTGTATTTCAGAGTTTCGTGAGAGGGTAGAAATTTGTATCCTATCTGGACCTAAAAGACAATCTTTTTATTGTAACTTTTATTTTTATGGGTTTCTTGGTATTGTGACATCATATGTAAAGGTTAGATTTAATTGTACTAATCGGATCCCGGGCCCGTCGACTGCAGAGGCCTGCATGCAAGCTTGGCGTAATCATGGTCATAGCTGTTTCCTGTGTGAAATTGTTATCCGCTCACAATTCCACACAACATACGAGCCGGAAGCATAAAGTGTAAAGCCTGGGGTGCCTAATGAGTGAGCTAACTCACATTAATTGCGTTGCGCTCACTGCCCGCTTTCCAGTCGGGAAACCTGTCGTGCCAGCTGCATTAATGAATCGGCCAACGCGCGGGGAGAGGCGGTTTGCGTATTGGGCGCTCTTCCGCTTCCTCGCTCACTGACTCGCTGCGCTCGGTCGTTCGGCTGCGGCGAGCGGTATCAGCTCACTCAAAGGCGGTAATACGGTTATCCACAGAATCAGGGGATAACGCAGGAAAGAACATGTGAGCAAAAGGCCAGCAAAAGGCCAGGAACCGTAAAAAGGCCGCGTTGCTGGCGTTTTTCCATAGGCTCCGCCCCCCTGACGAGCATCACAAAAATCGACGCTCAAGTCAGAGGTGGCGAAACCCGACAGGACTATAAAGATACCAGGCGTTTCCCCCTGGAAGCTCCCTCGTGCGCTCTCCTGTTCCGACCCTGCCGCTTACCGGATACCTGTCCGCCTTTCTCCCTTCGGGAAGCGTGGCGCTTTCTCATAGCTCACGCTGTAGGTATCTCAGTTCGGTGTAGGTCGTTCGCTCCAAGCTGGGCTGTGTGCACGAACCCCCCGTTCAGCCCGACCGCTGCGCCTTATCCGGTAACTATCGTCTTGAGTCCAACCCGGTAAGACACGACTTATCGCCACTGGCAGCAGCCACTGGTAACAGGATTAGCAGAGCGAGGTATGTAGGCGGTGCTACAGAGTTCTTGAAGTGGTGGCCTAACTACGGCTACACTAGAAGAACAGTATTTGGTATCTGCGCTCTGCTGAAGCCAGTTACCTTCGGAAAAAGAGTTGGTAGCTCTTGATCCGGCAAACAAACCACCGCTGGTAGCGGTGGTTTTTTTGTTTGCAAGCAGCAGATTACGCGCAGAAAAAAAGGATCTCAAGAAGATCCTTTGATCTTTTCTACGGGGTCTGACGCTCAGTGGAACGAAAACTCACGTTAAGGGATTTTGGTCATGAGATTATCAAAAAGGATCTTCACCTAGATCCTTTTAAATTAAAAATGAAGTTTTAAATCAATCTAAAGTATATATGAGTAAACTTGGTCTGACAGTTACCAATGCTTAATCAGTGAGGCACCTATCTCAGCGATCTGTCTATTTCGTTCATCCATAGTTGCCTGACTCCCCGTCGTGTAGATAACTACGATACGGGAGGGCTTACCATCTGGCCCCAGTGCTGCAATGATACCGCGAGATCCACGCTCACCGGCTCCAGATTTATCAGCAATAAACCAGCCAGCCGGAAGGGCCGAGCGCAGAAGTGGTCCTGCAACTTTATCCGCCTCCATCCAGTCTATTAATTGTTGCCGGGAAGCTAGAGTAAGTAGTTCGCCAGTTAATAGTTTGCGCAACGTTGTTGCCATTGCTACAGGCATCGTGGTGTCACGCTCGTCGTTTGGTATGGCTTCATTCAGCTCCGGTTCCCAACGATCAAGGCGAGTTACATGATCCCCCATGTTGTGCAAAAAAGCGGTTAGCTCCTTCGGTCCTCCGATCGTTGTCAGAAGTAAGTTGGCCGCAGTGTTATCACTCATGGTTATGGCAGCACTGCATAATTCTCTTACTGTCATGCCATCCGTAAGATGCTTTTCTGTGACTGGTGAGTACTCAACCAAGTCATTCTGAGAATAGTGTATGCGGCGACCGAGTTGCTCTTGCCCGGCGTCAATACGGGATAATACCGCGCCACATAGCAGAACTTTAAAAGTGCTCATCATTGGAAAACGTTCTTCGGGGCGAAAACTCTCAAGGATCTTACCGCTGTTGAGATCCAGTTCGATGTAACCCACTCGTGCACCCAACTGATCTTCAGCATCTTTTACTTTCACCAGCGTTTCTGGGTGAGCAAAAACAGGAAGGCAAAATGCCGCAAAAAAGGGAATAAGGGCGACACGGAAATGTTGAATACTCATACTCTTCCTTTTTCAATATTATTGAAGCATTTATCAGGGTTATTGTCTCATGAGCGGATACATATTTGAATGTATTTAGAAAAATAAACAAATAGGGGTTCCGCGCACATTTCCCCGAAAAGTGCCACCTGACGTCTAAGAAACCATTATTATCATGACATTAACCTATAAAAATAGGCGTATCACGAGGCCCTTTCGTC

**pUCC57 KRAS G12D plasmid**


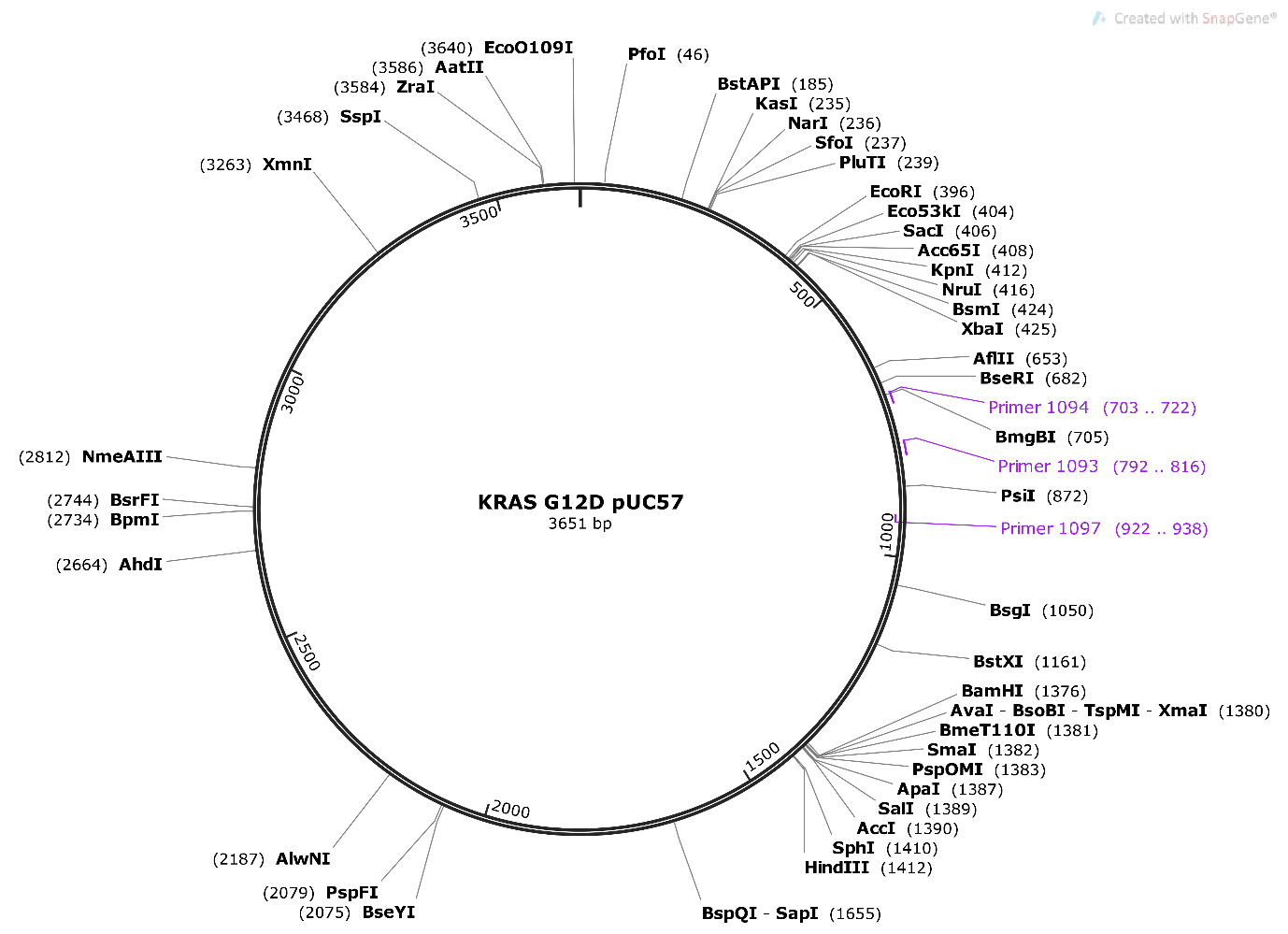


TCGCGCGTTTCGGTGATGACGGTGAAAACCTCTGACACATGCAGCTCCCGGAGACGGTCACAGCTTGTCTGTAAGCGGATGCCGGGAGCAGACAAGCCCGTCAGGGCGCGTCAGCGGGTGTTGGCGGGTGTCGGGGCTGGCTTAACTATGCGGCATCAGAGCAGATTGTACTGAGAGTGCACCATATGCGGTGTGAAATACCGCACAGATGCGTAAGGAGAAAATACCGCATCAGGCGCCATTCGCCATTCAGGCTGCGCAACTGTTGGGAAGGGCGATCGGTGCGGGCCTCTTCGCTATTACGCCAGCTGGCGAAAGGGGGATGTGCTGCAAGGCGATTAAGTTGGGTAACGCCAGGGTTTTCCCAGTCACGACGTTGTAAAACGACGGCCAGTGAATTCGAGCTCGGTACCTCGCGAATGCATCTAGATTCCACTAGGAAAACTGTAACAATAAGAGTGGAGATAGCTGTCAGCAACTTTTGTGAGGGTGTGCTACAGGGTGTAGAGCACTGTGAAGTCTCTACATGAGTGAAGTCATGATATGATCCTTTGAGAGCCTTTAGCCGCCGCAGAACAGCAGTCTGGCTATTTAGATAGAACAACTTGATTTTAAGATAAAAGAACTGTCTATGTAGCATTTATGCATTTTTCTTAAGCGTCGATGGAGGAGTTTGTAAATGAAGTACAGTTCATTACGATACACGTCTGCAGTCAACTGGAATTTTCATGATTGAATTTTGTAAGGTATTTTGAAATAATTTTTCATATAAAGGTGAGTTTGTATTAAAAGGTACTGGTGGAGTATTTGATAGTGTATTAACCTTATGTGTGACATGTTCTAATATAGTCACATTTTCATTATTTTTATTATAAGGCCTGCTGAAAATGACTGAATATAAACTTGTGGTAGTTGGAGCTGATGGCGTAGGCAAGAGTGCCTTGACGATACAGCTAATTCAGAATCATTTTGTGGACGAATATGATCCAACAATAGAGGTAAATCTTGTTTTAATATGCATATTACTGGTGCAGGACCATTCTTTGATACAGATAAAGGTTTCTCTGACCATTTTCATGAGTACTTATTACAAGATAATTATGCTGAAAGTTAAGTTATCTGAAATGTACCTTGGGTTTCAAGTTATATGTAACCATTAATATGGGAACTTTACTTTCCTTGGGAGTATGTCAGGGTCCATGATGTTCACTCTCTGTGCATTTTGATTGGAAGTGTATTTCAGAGTTTCGTGAGAGGGTAGAAATTTGTATCCTATCTGGACCTAAAAGACAATCTTTTTATTGTAACTTTTATTTTTATGGGTTTCTTGGTATTGTGACATCATATGTAAAGGTTAGATTTAATTGTACTAATCGGATCCCGGGCCCGTCGACTGCAGAGGCCTGCATGCAAGCTTGGCGTAATCATGGTCATAGCTGTTTCCTGTGTGAAATTGTTATCCGCTCACAATTCCACACAACATACGAGCCGGAAGCATAAAGTGTAAAGCCTGGGGTGCCTAATGAGTGAGCTAACTCACATTAATTGCGTTGCGCTCACTGCCCGCTTTCCAGTCGGGAAACCTGTCGTGCCAGCTGCATTAATGAATCGGCCAACGCGCGGGGAGAGGCGGTTTGCGTATTGGGCGCTCTTCCGCTTCCTCGCTCACTGACTCGCTGCGCTCGGTCGTTCGGCTGCGGCGAGCGGTATCAGCTCACTCAAAGGCGGTAATACGGTTATCCACAGAATCAGGGGATAACGCAGGAAAGAACATGTGAGCAAAAGGCCAGCAAAAGGCCAGGAACCGTAAAAAGGCCGCGTTGCTGGCGTTTTTCCATAGGCTCCGCCCCCCTGACGAGCATCACAAAAATCGACGCTCAAGTCAGAGGTGGCGAAACCCGACAGGACTATAAAGATACCAGGCGTTTCCCCCTGGAAGCTCCCTCGTGCGCTCTCCTGTTCCGACCCTGCCGCTTACCGGATACCTGTCCGCCTTTCTCCCTTCGGGAAGCGTGGCGCTTTCTCATAGCTCACGCTGTAGGTATCTCAGTTCGGTGTAGGTCGTTCGCTCCAAGCTGGGCTGTGTGCACGAACCCCCCGTTCAGCCCGACCGCTGCGCCTTATCCGGTAACTATCGTCTTGAGTCCAACCCGGTAAGACACGACTTATCGCCACTGGCAGCAGCCACTGGTAACAGGATTAGCAGAGCGAGGTATGTAGGCGGTGCTACAGAGTTCTTGAAGTGGTGGCCTAACTACGGCTACACTAGAAGAACAGTATTTGGTATCTGCGCTCTGCTGAAGCCAGTTACCTTCGGAAAAAGAGTTGGTAGCTCTTGATCCGGCAAACAAACCACCGCTGGTAGCGGTGGTTTTTTTGTTTGCAAGCAGCAGATTACGCGCAGAAAAAAAGGATCTCAAGAAGATCCTTTGATCTTTTCTACGGGGTCTGACGCTCAGTGGAACGAAAACTCACGTTAAGGGATTTTGGTCATGAGATTATCAAAAAGGATCTTCACCTAGATCCTTTTAAATTAAAAATGAAGTTTTAAATCAATCTAAAGTATATATGAGTAAACTTGGTCTGACAGTTACCAATGCTTAATCAGTGAGGCACCTATCTCAGCGATCTGTCTATTTCGTTCATCCATAGTTGCCTGACTCCCCGTCGTGTAGATAACTACGATACGGGAGGGCTTACCATCTGGCCCCAGTGCTGCAATGATACCGCGAGATCCACGCTCACCGGCTCCAGATTTATCAGCAATAAACCAGCCAGCCGGAAGGGCCGAGCGCAGAAGTGGTCCTGCAACTTTATCCGCCTCCATCCAGTCTATTAATTGTTGCCGGGAAGCTAGAGTAAGTAGTTCGCCAGTTAATAGTTTGCGCAACGTTGTTGCCATTGCTACAGGCATCGTGGTGTCACGCTCGTCGTTTGGTATGGCTTCATTCAGCTCCGGTTCCCAACGATCAAGGCGAGTTACATGATCCCCCATGTTGTGCAAAAAAGCGGTTAGCTCCTTCGGTCCTCCGATCGTTGTCAGAAGTAAGTTGGCCGCAGTGTTATCACTCATGGTTATGGCAGCACTGCATAATTCTCTTACTGTCATGCCATCCGTAAGATGCTTTTCTGTGACTGGTGAGTACTCAACCAAGTCATTCTGAGAATAGTGTATGCGGCGACCGAGTTGCTCTTGCCCGGCGTCAATACGGGATAATACCGCGCCACATAGCAGAACTTTAAAAGTGCTCATCATTGGAAAACGTTCTTCGGGGCGAAAACTCTCAAGGATCTTACCGCTGTTGAGATCCAGTTCGATGTAACCCACTCGTGCACCCAACTGATCTTCAGCATCTTTTACTTTCACCAGCGTTTCTGGGTGAGCAAAAACAGGAAGGCAAAATGCCGCAAAAAAGGGAATAAGGGCGACACGGAAATGTTGAATACTCATACTCTTCCTTTTTCAATATTATTGAAGCATTTATCAGGGTTATTGTCTCATGAGCGGATACATATTTGAATGTATTTAGAAAAATAAACAAATAGGGGTTCCGCGCACATTTCCCCGAAAAGTGCCACCTGACGTCTAAGAAACCATTATTATCATGACATTAACCTATAAAAATAGGCGTATCACGAGGCCCTTTCGTC

**Supplementary Figure 5**

Amplified linear gene spike-in fragments:

**GNP1 –** 337 bp

Forward primer: GCACCGTTAGCAACGGAAAG

Reverse primer: GGTTCAAAGGTGTCGTTGCC

GCACCGTTAGCAACGGAAAGAACAGACAACAGGATGACAGCGTTTATGAAATGAGGAACCACACGAACACCATGAGAGGAGACAGCGATGACGTAGGGCGACGCTTTAGTGGCTGAACCTGCGGCCCCTAGCAATTGATCTGAGGTGTAAGGTACAAGGAAACCAACTAACGTTAAAGACGCCAAGAACACAAACAGAATTCTATAAATCATTTTCTTTGCCGCCGATGGAATAGCCTTTCTTGGATTGGATTGTTCACTGGCAGTCATAGCCAGCTGTTCACTCATACCAAAGGCGAACGCTGCTGTGACAAATGTGGCAACGACACCTTTGAACC

**AGP1** – 820 bp

Forward primer: GCACAGAAGGCAATAACGGC

Reverse primer: GTTTTGGGTTTGCAGTCGCT

GCACAGAAGGCAATAACGGCAAACAATGCAGAAACACCCATGGCAATCAATGGTCTACCGGCTCTGTCGATGTAGGAGAAAACCTTAGGAGCGTAACCTTGCTCGGATAGAGTTAAAAATAAACGAGCACTGGAGTAGAAGGAGGAGTTAGCCATGGACAGCACGGAAAGTAGAATAACGGCGTTAATGAAGTGTGGGACGACACGGACACCGTGGGATGCAACAGCAATGACATATGGCGAGGCTTTAGTACCACCACCGGTAGAACCCAATAATTGATCGGAATTGTATGGCACCAAGAAACCCAGTAGAATAATGGTAGCCAAGAATAGGAATAAGATTCTGTAGATCATTTGTTTGGCCGCACCTGGAATGGCCTTTCTTGGATTAGATTGTTCTGCAGTGGTGATGGCAATAAACTCTGAACCACCAAAGGCAAAAGCAGCAGTCACTAATGTTGCAGCAACACCTTTAAATCTGTCAATGGCATGTTTACCATTGAAAGCGCCCGGATCGTGCCAGTATTTACCACCAATAAAACCATCATTACCAGCGCCACCAACATCGATGATAATACCAAGAATGAAGAACCCAGTCATCATCAAAATTTTGCAACAGTTGAAGAAGAACTCAGCTTCTGCATAACCACGAGCACCGAAAATATTAATAGTAATCACCAAAACATAGAAAATAATGACGAACACATCCGGGTTCACAGATGTCGTCCAATATTTGATAGTCATGGATGCGGTCACCAATTCCAGAGGACACACACACAGCCATTGCAAACAATAAACCCAAGCGACTGCAAACCCAAAAC

**ACT1** – 1409 bp

Forward primer: TGGATTCTGGTATGTTCTAGC

Reverse primer: TGGACCACTTTCGTCGTATTC

TGGACCACTTTCGTCGTATTCTTGTTTTGAGATCCACATTTGTTGGAAGGTAGTCAAAGAAGCCAAGATAGAACCACCAATCCAGACGGAGTACTTTCTTTCTGGAGGAGCAATGATCTTGACCTTCATGGAAGATGGAGCCAAAGCGGTGATTTCCTTTTGCATTCTTTCGGCAATACCTGGGAACATGGTGGTACCACCGGACATAACGATGTTACCGTATAATTCCTTACGGACATCGACATCACACTTCATGATGGAGTTGTAAGTAGTTTGGTCAATACCGGCAGATTCCAAACCCAAAACAGAAGGATGGAACAAAGCTTCTGGGGCTCTGAATCTTTCGTTACCAATAGTGATGACTTGACCATCTGGAAGTTCGTAGGATTTTTCAATTGAAGAAGATTGAGCAGCGGTTTGCATTTCTTGTTCGAAGTCCAAGGCGACGTAACATAGTTTTTCCTTGATGTCACGGACAATTTCTCTTTCAGCAGTGGTGGAGAAAGAGTAACCACGTTCACTCAAGATCTTCATCAAGTAGTCAGTCAAATCTCTACCGGCCAAATCGATTCTCAAAATGGCGTGAGGTAGAGAGAAACCAGCGTAAATTGGAACGACGTGAGTAACACCATCACCGGAATCCAAAACAATACCAGTAGTTCTACCGGAAGAGTACAAGGACAAAACGGCTTGGATGGAAACGTAGAAGGCTGGAACGTTGAAAGTTTCAAACATAATTTGAGTCATCTTTTCTCTGTTTGATTTAGGGTTCATTGGAGCTTCAGTCAAAAGAACAGGGTGTTCTTCTGGGGCAACTCTCAATTCGTTGTAGAAGGTATGATGCCAGATCTTTTCCATATCGTCCCAGTTGGTGACAATACCGTGTTCAATTGGGTAACGTAAAGTCAAGATACCTCTCTTGGATTGAGCTTCATCACCAACGTAGGAGTCTTTTTGACCCATACCGACCATGATACCTTGGTGTCTTGGTCTACCGACGATAGATGGGAAGACAGCACGAGGAGCGTCGTCACCGGCAAAACCGGCTTTACACATACCAGAACCGTTATCAATAACCAAAGCAGCAACCTCTAAACATATAATATAGCAACAAAAAGAATGAAGCAATCGATGTTAGTACATGAGACTTAGTAACAGTAGCAAATAAAAGAAGAATATCACTATCACTTATCACGAAAATATTTCAGTTTTTTTTTTTTTTCCAAATTTCAAGCCCCTATTTATTCCAATAATATCGTGGTTATTACAGATCAGTCAATATAGGAGGTTATGGGAGAGTGAAAAATAGTAAAAAAAGGTAAAAGAGAAATCTCTCGAGCAATTGGGACCGTGCAATTCTTCTTACAGTTAAATGGGATGGTGCAAGCGCTAGAACATACCAGAATCCA

**BCP1 –** 2716 bp

Forward primer: TCGGATAGCCTCTGGTTAGG

Reverse primer: TCAGTACAGTTGCGGTGGAC

TCGGATAGCCTCTGGTTAGGTACATTTATTAAATTTACATCCTTCAATTTCTTTTTTGTTATGGGAGTCGCAGCTGTATGTAGGAGCTTCGGCAGTGACGCCTCGTCAAACCTTATACCCCTTCGTATATCGTTTTTCTTTAAAAATGCATCTATTTCCTTCCGAAGTTTGTTTTCATCTGTTAACTCTAGCAGCTGGTTCAAGCGGGATACGCAGTATAGTTCGGTCAGTTTTCTATCTCTTTCATCAATTAAGTCATCTGCCTTAGGAAATTTACTTCTATCACTGCTCTGGTCCTCACTGAGTGATGCTGAATTAGGCACTGCTGAACTTGGACGTGAGGACATCTTTGAGGCGGAAACTGTACTTTTGATTATGATGTCTCTAAATAATCTTTATATTCTATCTGTTCGGCGGCTAACGCCTACGGGTAATAGCCGGCCTTTGAAAATTTCGCGGCTGCAGCAAGTACGCAAAAATCCTTATCCCTTCTAACGGTTCGAAAGGGGGAAGAACCAGAAATTTTGTAGTTACGCCGCAAATACCTGTATGCCCCGGCTCAAAATGCTCAACACTTATATGCTGACTATGTTCTATAAGGCTCAGGGTGAATGACCTTTCTTGAATTACAATCTGATTTCACTTTGCACAATATATACTATGTATTTTTATTGGAGCACACGACATTAAAAAGATGATCGCCCAGAAAAATTTTCAGCGGTGGCGGATTAATTTTTACTGTCCTCGGCAGCCCAAAATTATCCGTGTAGGATGACATAAAGGCTCAATGGCCGCTGCTGCTTCGTCTGTTAATAATATACAAATTGCACTAACTGTGCAGGGCAGTGATCGCAACTATTTACTTTTCGTAAAATTACCAAGTGGAAATTTCTGTTTCCAATTCATCGATACTTTTGACAAGGCCTTCGTGATCAAGAATCATGTATGAGCTGATAACACCCTTTTTAGCTTCTGATTCGAAGTGAATCTTGGCATATTTTTCAAAAAATCGGTCCTCCTCATGAAAGTAGTCTACTTCATCGGCCTTCACCCTTTTTTTGGATCTTTCGTCTTTGTTTTTATTCCTCTTACCAGAGTCGGTATCATCGTCAGTGTCAAAATTTACTTCATACTTCCTGGTGACGATGATATAGAAGTCATAATGTTTGTCATCGCCAAGTGCCGTGGCAACATCCTCCAACGTAATCTTGTATAAAGGTGGAACGACTTCCGGTGGCATATTAATCAGCCTTTCACTGAGAACCAAAGCACAATTTTTATTACCACTATCAATCATAGTTTTGAAGAAAGTGGAAAGTCTCATGTCCACTTTTTGTAAATATTTGACATAATCACTTAGATGATTAGCTTTGAAATCAACGAATGAAAGAAAACAGTATGGATCAGATTCTTTGCCGTCTGTTTTAATTGTGGTGGTTGGGGAACCTAGGATCAAATCTGCCAAGCTGCTTAGTTGAATCCTGGTACTTTCTTGAGGACCAAATAACTGACGCAGTAAATTCTTTAAAGCATGAAAATCAACTTCTGGGTTACCACCGAAAAAATCGAAGTCAATATTTACGATCTCTTCTTCTCCATTTTGCTCTTCTTCATTTTCTGAATCGGTGCTACTAATGTCAATTTCAGACTCGTCACTGCCATTTTCTTCCTCCACATTTTTTCTCTTCCTATTTTTTAAATCATTTAATTTGATAGCTTGAACCATATTTGTGAGTTAATTAGCAATCCTATGAAAATGTGGGCCTCATGAACAATCTAAATGATAAAATTATAAGGACATAATAGATAATATATTGACAACTCTTTTTCAGATATTTTTTCTACCTCAATCTCATCGCGAGGAATATAGGCACGGAAAGAGAAACTCGTATAAAGTGACCACGGATATCGAGAATTTGATAATTTACCCTTAACTGAAGTTATGGCACAATATCTGTATAAATTTTAATACATAATTTGCGGCAGGAATAACTGCAACAATACTCAAAGTTATTAGTGCTAGCCAGGCGATATTTTACATTAGGCTATTTTATTCCTTTTTCATTTCAATAAAAAAAGATAGTTTTTTTCGTCAACAATAGTTCAATGTCACAAATTGTATTTATTACGTAAAGTCCATCTCATGACAGGTATTCGAGTGTTAATAGTCCAGCACTATTAGAAAATGCATAACATTTGCCTCCAGCGCTCGTTTCATTCCATTTTGTACATGTTATATTAATGCCGTGCGCATCTATTTTAGCTTTACTAACATCGTTGACCGTCAGCGGATACACTTCATAAGAACTATCAATTCTATATTTGTCATCTTTTATGCTATAATCCCATTTCCAAAGCCTTAATGACTTTTGAGTTGCTGAACTGTTTTTAATGCCATGCAAAAGGCGCCTAGCTGCGTTGGTTATAATAAGACTTCCATCTGCTGATCCAGCAAGGACCATTGGATGTAATCTTGACACCCCAATGGCCGTTATGGTCGTCTCTCGCGACACTAAAGGATGAACTGCGAAAGCAGCCCTAGACGGCACAGCCCTTAAGGAACTTGCACCATCTGAGTAAATATACGAGTAGATTTGAGGACAATAAACAACTGGAACCAAATTACTTCCCCTGAATCTGGACACCGTGGTCTTTGTTGTAGCAATGTCTTTTGGATTGAAAATGTAAAAGTAGCCGTCCACCGCAACTGTACTGA
